# Transcriptional activation domains interact with ATPase subunits of yeast chromatin remodelling complexes SWI/SNF, RSC and INO80

**DOI:** 10.1007/s00294-024-01300-x

**Published:** 2024-09-05

**Authors:** Eva-Carina Wendegatz, Maike Engelhardt, Hans-Joachim Schüller

**Affiliations:** 1 Center for Functional Genomics of Microbes, Institut Für Genetik Und Funktionelle Genomforschung, Universität Greifswald, Felix-Hausdorff-Strasse 8, 17487 Greifswald, Germany; 2 Present Address: Cheplapharm, Greifswald, Germany

**Keywords:** *Saccharomyces cerevisiae*, Activation domains, Chromatin remodelling complex, Swi2, Sth1, Ino80, Activator binding domain

## Abstract

Chromatin remodelling complexes (CRC) are ATP-dependent molecular machines important for the dynamic organization of nucleosomes along eukaryotic DNA. CRCs SWI/SNF, RSC and INO80 can move positioned nucleosomes in promoter DNA, leading to nucleosome-depleted regions which facilitate access of general transcription factors. This function is strongly supported by transcriptional activators being able to interact with subunits of various CRCs. In this work we show that SWI/SNF subunits Swi1, Swi2, Snf5 and Snf6 can bind to activation domains of Ino2 required for expression of phospholipid biosynthetic genes in yeast. We identify an activator binding domain (ABD) of ATPase Swi2 and show that this ABD is functionally dispensable, presumably because ABDs of other SWI/SNF subunits can compensate for the loss. In contrast, mutational characterization of the ABD of the Swi2-related ATPase Sth1 revealed that some conserved basic and hydrophobic amino acids within this domain are essential for the function of Sth1. While ABDs of Swi2 and Sth1 define separate functional protein domains, mapping of an ABD within ATPase Ino80 showed co-localization with its HSA domain also required for binding actin-related proteins. Comparative interaction studies finally demonstrated that several unrelated activators each exhibit a specific binding pattern with ABDs of Swi2, Sth1 and Ino80.

**Supplementary Information:**

The online version contains supplementary material available at 10.1007/s00294-024-01300-x.

## Introduction

Initiation of transcription in eukaryotes depends on a large number of general factors which together with RNA polymerase II finally form a pre-initiation complex (PIC) at the basal promoter of a gene to be activated (reviewed by Malik and Roeder 2023). However, nucleosomal organization of eukaryotic chromatin may negatively influence access of factors such as TFIID to DNA (Hu et al. 2014), requiring efficient mechanisms to allow a transition from “closed” to “open” chromatin at regulated genes. This may be achieved by covalent modification of histones (Chou et al. 2023) and/or by ATP-dependent chromatin remodelling complexes (SWI/SNF, RSC, INO80 and others) which are able to mobilize nucleosomes by sliding but can also eject nucleosomes from their position or change their composition by histone dissociation (Clapier et al. 2017; Eustermann et al. 2024). As a result of their enzymatic function, nucleosome-depleted regions are generated at basal promoters which are now more easily accessible for general factors finally initiating gene expression.

Chromatin remodelling complexes (CRC) were identified genetically by isolation of mutants with pleiotropic activation defects affecting structural genes of unrelated function (e. g. *swi2* = *snf2*: defective for *HO* induction and thus unable to switch mating types, deficient for *SUC2* derepression and failure to utilize sucrose as the sole carbon source; Stern et al. 1984; Neigeborn and Carlson 1984). Isolation of suppressor mutations relieving phenotypes of *swi*/*snf* mutants revealed qualitative or quantitative alterations in histones, providing evidence that the products of *SWI*/*SNF* genes may help to overcome the general negative influence of chromatin on transcription (Winston and Carlson 1992). *SWI2* has been shown to encode a protein with multiple functions among which its DNA-dependent ATPase/translocase domain (containing two RecA core domains) is required for nucleosome contacts and subsequent sliding along DNA by repeated binding and hydrolysis of ATP, breaking interactions between histones and DNA (Clapier et al. 2017). Swi2 turned out as the enzymatic core of the SWI/SNF protein complex (containing 12 subunits such as Swi1, Snf5 and Snf6) the structure of which has been solved in the nucleosome-bound and nucleosome-free state by cryo-electron microscopy (cryo-EM; Han et al. 2020; Wang et al. 2020). A bromodomain at the C-terminus of Swi2 intensifies promoter occupancy of SWI/SNF by binding to acetylated lysines of histones H3 and H4 (Hassan et al. 2007). Swi2 also contains the evolutionary conserved SnAC domain (Snf2 ATP Coupling) required for its ATPase function as well as AT-hook domains mediating unspecific DNA-binding to AT-rich sequences (Sen et al. 2011). Finally, the HSA domain (helicase-SANT-associated) of Swi2 binds actin and nuclear actin-related proteins Arp7 and Arp9 (Szerlong et al. 2008). Comparative transcript studies of *SWI2* wild-type and mutant strains using microarrays revealed that expression of about 1% of protein-encoding genes in yeast was significantly Swi-dependent (Sudarsanam et al. 2000). SWI/SNF subunits are evolutionary conserved in all eukaryotic organisms and it has been estimated that more than 20% of all human malignant tumors show mutations in subunits of SWI/SNF-related complexes BAF and PBAF (Biegel et al. 2014).

Importantly, Sth1 (Snf two homolog) has been identified as a strongly conserved (and more abundant) yeast paralog of Swi2 and later characterized as the enzymatic core of a second CRC, designated RSC (remodels the structure of chromatin; Cairns et al. 1996). However, in contrast to subunits of SWI/SNF, Sth1 and most of the 15 remaining subunits of RSC are essential for cellular viability. Depletion experiments with a Sth1 variant containing an inducible degron provided convincing evidence that RSC plays an outstanding role in the establishment and maintenance of nucleosome-free regions (NFR) in AT-rich promoter sequences, flanked by precisely positioned upstream and downstream nucleosomes (Hartley and Madhani 2009; Yen et al. 2012; reviewed by Lorch and Kornberg 2017). The structure of RSC bound to a nucleosome has been solved by cryo-EM (Patel et al. 2019; Ye et al. 2019; Wagner et al. 2020).

INO80 as an additional pleiotropic CRC was initially identified by characterization of the *ino80* mutation which is auxotrophic for inositol due to strongly reduced expression of *INO1* and other genes of phospholipid biosynthesis (Ebbert et al. 1999). Similarly, *INO80* stimulates activation of genes required for phosphate acquisition as well as for utilization of non-fermentable carbon sources and is also involved in replication and in repair of DNA double-strand breaks. Ino80 belongs to a different subclass of remodelling enzymes with an ATPase/translocase domain containing an extended linker region between RecA lobes and forms a complex with 14 additional subunits such as actin as well as Arp4, Arp5 and Arp8 (Shen et al. 2000; 2003). CRC INO80 positions + 1 nucleosomes close to transcriptional start sites by sliding and also influences the promoter distribution of histone variant H2A.Z by its histone-exchange activity. While the INO80-related CRC SWR1 replaces H2A-H2B dimers in nucleosomes with H2A.Z-H2B, INO80 can inversely exchange H2A.Z-H2B with free H2A-H2B (Papamichos-Chronakis et al. 2011). Recently, the hexasome as a subnucleosomal particle devoid of a H2A-H2B dimer has been described as the genuine substrate of CRC INO80 which can slide hexasomes much more efficiently than complete nucleosomes (Hsieh et al. 2022; Zhang et al. 2023).

Functional aspects of sliding/ejecting nucleosomes by ATPase domains of CRC and binding of actin-related proteins to their HSA domains have been intensively studied. However, less is known about mechanisms how transcriptional activation domains (TADs) of activator proteins contact CRC and may recruit them to specific genes to be transcribed. Although TADs cannot be identified by simple consensus motifs, acidic amino acids (especially aspartic acid) and bulky hydrophobic residues have been shown to be critical for function, allowing prediction of TADs by bioinformatic strategies (such as ADprep; Erijman et al. 2020). Importantly, TADs are considered as intrinsically disordered, becoming structured upon interaction with activator binding domains (ABDs). There is evidence that TAD-ABD interactions result in local phase separation and formation of so-called condensates (Hahn 2018). Several subunits of SWI/SNF such as Swi1, Swi2, Snf5 and Snf6 can interact with activators Gcn4, VP16 and Hap4 (Neely et al. 1999; 2002; Yudkovsky et al. 1999) and activator binding domains have been mapped within Swi1 and Snf5 (Prochasson et al. 2003). However, it remains unclear how activators mediate access of complexes RSC and INO80 to their target genes. Transcriptional activator Ino2 of *S. cerevisiae* contains two separate activation domains (TAD1, TAD2) and stimulates expression of phospholipid biosynthetic genes when inositol as a metabolic precursor becomes limiting (Schwank et al. 1995; Dietz et al. 2003). We have previously shown that these TADs can recruit various coactivator subunits of general transcription factors TFIID and TFIIA (Taf1, Taf4, Taf6, Taf10, Taf12, Bdf1 and Toa1; Hintze et al. 2017; Engelhardt et al. 2023), increasing their presence at promoters with binding sites of Ino2 and its partner protein Ino4. While these interactions should stimulate formation of the transcriptional pre-initiation complex, it remained unclear whether Ino2 can also affect chromatin organization, preventing repression by positioned nucleosomes. In this work we demonstrate that Ino2 (as well as a number of unrelated activators) can indeed interact with ATPases Swi2, Sth1 and Ino80 and SWI/SNF subunits Swi1, Snf5 and Snf6. Characterization of activator binding domains (ABDs) within these coactivator proteins of CRCs SWI/SNF, RSC and INO80 is important for a better understanding of their TAD-dependent promoter recruitment and/or the stimulation of their remodeling activity. Consequently, ABDs identified in this work were studied for their functional importance in vitro and in vivo by constructing deletion variants and introducing missense mutations.

## Materials and methods

### Yeast and bacterial strains, media and growth conditions

Complete genotypes of all *S. cerevisiae* strains used in this work are compiled in Table S1 (Supplementary material). Strains JS94.8-12 and EWY4 (*swi2*Δ) were derived from CY57 (Peterson and Herskowitz 1992) by multiple back-crossings with strains of our genetic background. The mutant allele *sth1*Δ*::HIS3* in strain KSY1 was introduced by gene disruption and led to deletion of Sth1 amino acids 145–1216. Strain YAB1 (*ino80*Δ) has been described (Ebbert et al. 1999). For in vitro interaction assays, yeast protein extracts were prepared from transformants of *S. cerevisiae* strain C13-ABY.S86 deficient for major vacuolar proteinases (*pra1 prb1 prc1 cps1*; Hintze et al. 2017). To prepare bacterial protein extracts, *E. coli* strain BL21-CodonPlus(DE3)-RP (Stratagene/Agilent) containing additional tRNA genes was used. Chemically defined synthetic media for growth of transformants upon variation of carbon sources and availability of phospholipid precursors inositol/choline have been described (Schwank et al. 1995; Ebbert et al. 1999).

### Plasmid constructions

*Escherichia coli* expression plasmids encoding IPTG-inducible fusions of glutathione-S-transferase (GST) with transcriptional activation domains of Ino2, Gal4, Gcn4, Rap1, Aro80, Leu3 and Swi5 were used for in vitro-interaction experiments (Hintze et al. 2017; Engelhardt et al. 2023). HA_3_-fusion proteins were synthesized in *S. cerevisiae*, using multi-copy plasmids with the *MET25* promoter (derepressible in the absence of methionine; p426-MET25HA, Mumberg et al. 1994). For bacterial synthesis of HA_3_-tagged proteins, expression plasmids derived from pASK-IBA5-HA3 (tet promoter-operator, inducible with anhydrotetracycline; IBA, Göttingen, Germany) were used. Length variants of coactivator-encoding genes were generated by PCR-amplification with position-specific primers. Internal deletions were introduced by inverse PCR, using primers flanking the sequence to be deleted and template plasmids with longer gene insertions (Imai et al. 1991).

To introduce missense mutations into coding regions of *SWI2* and *STH1*, the QuikChange site-directed mutagenesis kit (Thermo Fisher) was used. Gene-specific mutagenic primers replaced selected natural codons by alanine-specific codons. To confirm the authenticity of mutational variants, missense and deletion constructs were verified by DNA sequencing (LGC Genomics, Berlin, Germany). Genotypes of expression plasmids and gene-specific primers used for their construction are shown in Tables S2 and S3, respectively (Supplementary material).

### In vitro interaction assays

In vitro interaction assays were performed as described by Hintze et al. (2017). In brief, GST fusions were released from *E. coli* by sonication and immobilized on glutathione (GSH) sepharose beads. Induction of *GST* fusion genes with IPTG and affinity purification of GST-TAD fusions containing TADs of Ino2 (TAD1, TAD2), Aro80, Gal4, Gcn4, Leu3, Rap1 and Swi5 is shown in Fig. S5 (Supplementary material). GST enzyme assays were performed to ensure that similar amounts of GST fusions were used as “bait” proteins. A detailed description of the GST enzyme assay is available in the Supplementary material (Experimental procedure 1). Fusion proteins bound to GSH sepharose were incubated with total protein extracts from transformants of yeast or *E. coli* containing HA fusions of Swi1, Swi2, Swi3, Snf5, Snf6, Sth1 or Ino80, using incubation buffer SA-125 (20 mM HEPES, pH7.6; 1 mM dithiothreitol (DTT), 1 mM EDTA, 125 mM K-acetate, 20% glycerol, 1% NP-40; Kadosh and Struhl 1997). Protein extracts from yeast transformants were prepared by mechanical agitation in the presence of zirconia beads and from *E. coli* transformants by sonication, respectively. Following elution with an excess of free GSH, proteins were separated by SDS/PAGE, transferred to a PVDF membrane and incubated with anti-HA-peroxidase conjugate (monoclonal antibody 12CA5 conjugate; Sigma-Aldrich). To visualize antibody-bound HA-tagged proteins, the membrane was treated with a POD chemiluminescent substrate. The resulting luminescence was detected with a digital imager (ChemoStar, Intas, Göttingen, Germany). All interaction experiments were performed at least twice; minimal domains mapped were routinely confirmed three times.

### Plasmid shuffling

While viable mutants *swi2* and *ino80* could be used for testing functional complementation with variants of *SWI2* and *INO80*, respectively, we performed plasmid shuffling experiments (Sikorski and Boeke 1991) to investigate variants of the essential *STH1* gene. *STH1* together with its native control region was amplified by PCR and inserted into *ARS CEN URA3* vector YCplac33 (Gietz and Sugino 1988). After transformation of the resulting rescue plasmid pKS2 into wild-type strain JS91.15-23 (Schwank et al. 1995), most of the coding region of the chromosomal *STH1* gene was deleted (amino acids 145–1216), using a *sth1*Δ*::HIS3* gene disruption cassette (plasmid pKS10). The resulting strain KSY1 was then transformed with single-copy *ARS CEN LEU2* plasmids derived from YCplac111 (Gietz and Sugino 1988), containing deletion or missense variants of *STH1*. Functional complementation of the *sth1* null mutation by *STH1* variants was finally assayed by incubating transformants on synthetic medium containing 5-fluoroorotic acid (FOA), allowing counter-selection against rescue plasmid pKS2.

## Results

### Interaction of ATPase Swi2 with transcriptional activation domains

**Fig. 1:**
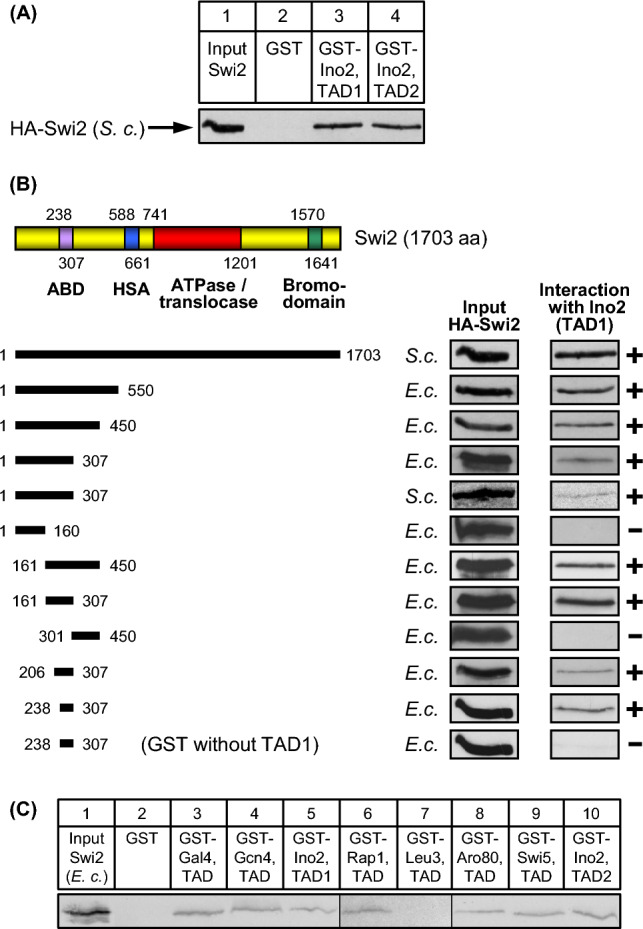
In vitro interaction assays (GST pull-down) with length variants of Swi2 and various activation domains. **A** Swi2 interacts with both Ino2 activation domains. Fusion proteins GST-Ino2 TAD1 (aa 1–35; encoded by pSH117) and GST-Ino2 TAD2 (aa 101–135; pSH 118) were synthesized in *E. coli*, immobilized on GSH sepharose and incubated with yeast protein extracts containing epitope-tagged Swi2 (full-length; synthesized by yeast expression plasmid pES17). **B** Mapping of Swi2 domains interacting with TAD1 of Ino2. The following expression plasmids were used to synthesize HA-Swi2 variants in *E. coli* strain BL21 CodonPlus: pMG142 (aa 1–550), pMG140 (aa 1–450), pIB2 (aa 1–307), pIB1 (aa 1–160), pIB3 (aa 161–450), pIB4 (aa 161–307), pIB5 (aa 301–450), pIB9 (aa 206–307) and pIB12 (aa 238–307). Length variant aa 1–307 was also synthesized in *S. cerevisiae*, using yeast expression plasmid pECW41. ABD, activator binding domain; HSA, helicase/SANT-associated domain. ATPase RecA subdomains are not shown individually. Functional domains SnAC (Snf2 ATP coupling, aa 1302–1369) and AT-hook (aa 1446–1458 and aa 1518–1530) are omitted from the figure. **C** TADs of various unrelated transcriptional activators interact with Swi2. GST fusions of Ino2 TAD1 (aa 1–35; pSH117), Ino2 TAD2 (aa 101–135; pSH118), Gal4 TAD (aa 768–881; pES20), Gcn4 TAD (aa 9–172; pKH60), Leu3 TAD (aa 841–886; pES5), Rap1 TAD (aa 630–671; pLJ6), Aro80 TAD (aa 846–950; pMG50) and Swi5 TAD (aa 1–85; pDG1) were synthesized in *E. coli*, immobilized on GSH sepharose and incubated with bacterial protein extract containing epitope-tagged Swi2 length variant aa 161–307 (pIB4). Empty GST plasmid pGEX-2TK was used as a negative control

Null mutant alleles of *SWI2* encoding the ATPase subunit of the SWI/SNF complex cause severe loss of *INO1* transcription and thus auxotrophy for inositol, among other pleiotropic phenotypes (Peterson et al. 1991; Peterson and Herskowitz 1992). Thus, Swi2 may be a candidate for interaction with transcriptional activation domains of Ino2 triggering stimulated expression of *INO1* under inositol-limiting conditions. Swi2 has been previously identified as a target of activation domains from the ubiquitous activator VP16 and activator Gcn4, stimulating expression of amino acid biosynthetic genes (Neely et al. 2002). However, a functional domain within Swi2 mediating activator interaction has not been identified, possibly due to stability problems with truncated protein variants. To investigate whether Swi2 can also interact with activation domains of Ino2, we epitope-tagged the coding region of *SWI2* and used yeast protein extracts containing HA-Swi2 for in vitro-interaction studies (GST pull-down experiments) with immobilized GST fusions of TAD1 (aa 1–35) and TAD2 of Ino2 (aa 101–135). As is shown in Fig. 1 A, both activation domains are able to bind Swi2. For a precise mapping of the activator-binding domain within Swi2, various length variants of *SWI2* were fused with HA and assayed for synthesis of stable HA-Swi2 proteins in yeast and/or *E. coli*. With the exception of variant aa 1–307 which could be obtained from both organisms, only bacterially produced length variants turned out to be stable and were subsequently used for pull-down studies (Fig. 1B). We focused on Swi2 truncations covering its N-terminus because several functional domains such as HSA, ATPase/translocase, SnAC, AT-hook and bromodomain have been already identified in the remaining part of the protein. Indeed, an N-terminal length variant (aa 1–307) synthesized in yeast and *E. coli* could interact with Ino2 TAD1. Further truncations finally enabled us to identify a 70 aa length variant of Swi2 (aa 238–307) as an activator-binding domain (ABD, Fig. 1B) which could not only interact with both TADs of Ino2 but also with TADs of unrelated activators Gal4, Gcn4, Rap1, Aro80 and Swi5 (Fig. 1C). Nevertheless, we cannot completely exclude that Swi2 contains an additional ABD within regions of the protein which we have covered less detailed by our deletion analysis.

**Fig. 2:**
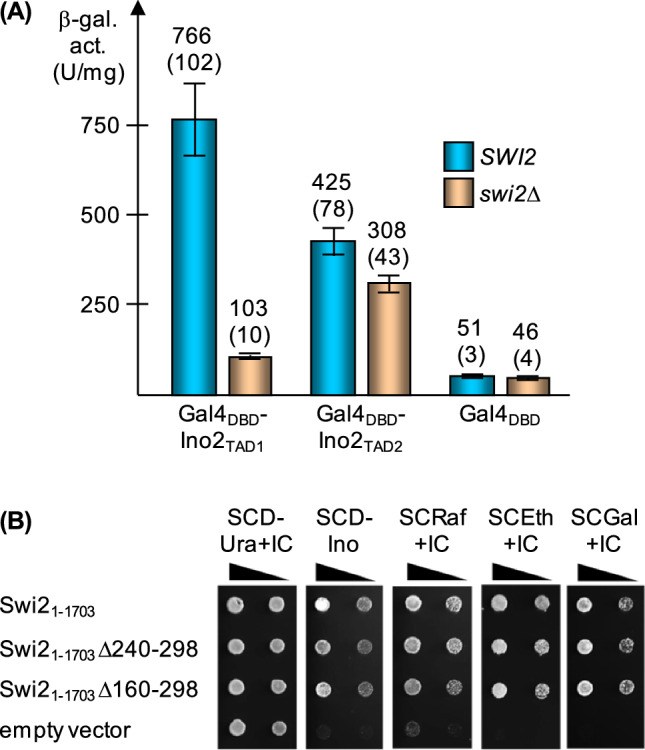
Influence of *SWI2* on gene activation by Ino2. **A** Expression of a *GAL1-lacZ* reporter gene in the presence of Gal4_DBD_-Ino2 hybrid activators. Isogenic strains EWY3 (*SWI2 gal4*Δ) and EWY4 (*swi2*Δ *gal4*Δ) were doubly transformed with reporter plasmid pKH29 (*GAL1-lacZ*) and effector plasmids pSG14 (*GAL4*_DBD_-*INO2*_TAD1_), pSG16 (*GAL4*_DBD_-*INO2*_TAD2_) and pGBD-C1 (*GAL4*_DBD_, negative control, basal expression), respectively. Transformants were grown in selective medium under derepressing conditions (0.2% glucose + 1% lactate) until mid-log phase. Specific activities of β-galactosidase are given in U per mg of protein. Activation in vivo was assayed by performing three independent transformations of both strains, using four colonies for each repetition (12 individual enzyme assays). Standard deviations are indicated by error bars and given in parentheses. **B** Functional complementation of *swi2* mutant phenotypes by *SWI2* variants devoid of sequences encoding the activator binding domain (ABD). *S. cerevisiae* strain JS94.8-12 (relevant genotype: *ura3 swi2*Δ*::HIS3*) was transformed with plasmids pECW24 (*ARS CEN URA3 MET25*_Pr_-*SWI2*; wild-type), pECW28 (*MET25*_Pr_-*SWI2*Δ240-298), pECW30 (*MET25*_Pr_-*SWI2*Δ160-298) and p416-MET25 (empty vector), respectively. Serial strain dilutions were spotted on the media shown and incubated for one (SCD-Ura + IC), two (SCD-Ino, SCRaf + IC and SCGal + IC) or three days (SCEth + IC). IC, inositol + choline; Ino, inositol; Raf, raffinose; Eth, ethanol; Gal, galactose

As an in vivo assay for Swi2-dependent gene activation, we used Gal4_DBD_-Ino2 TAD fusions in combination with a *GAL1-lacZ* reporter gene and compared reporter gene expression in isogenic *SWI2* and *swi2* strains. As is shown in Fig. 2A, TAD1 and TAD2 mediated strong activation of the reporter gene when *SWI2* is functional. Activation by TAD1 was substantially weakened in the *swi2* null mutant (to 13.4%) while TAD2 was only slightly affected (reduction to 72.5%). Although both TADs of Ino2 were able to interact with Swi2, TAD1 was significantly more dependent on Swi2 than TAD2.

To characterize the Swi2 ABD in more detail, we introduced missense mutations at selected positions into the ABD coding region and comparatively investigated whether the in vitro interaction of the Swi2 variants obtained with Ino2 TAD1 is compromised. We compared Swi2 sequences from various yeasts (Supplementary Fig. S1) and selected nine combinations of two, three or four conserved amino acids (F248 T249, E251 Q252 S253, L256 K257 L263 K264, I260 T261, L266 V267 N268, K270 P271, V279 I280 Q281, H286 P287 F290 K291 and R292 M293) which were replaced by alanine residues using site-directed mutagenesis. All variants of Swi2 ABD could be efficiently synthesized in *E. coli* but none of them was significantly impaired for in vitro interaction with TAD1 of Ino2 (Supplementary Fig. S2). We assume that a considerable degree of functional redundancy may exist among critical amino acids as we have shown previously for interaction of Ino2 with TFIID subunit Taf12 (Hintze et al. 2017).

To investigate whether the ABD of Swi2 is important for the function of the SWI/SNF complex we constructed two *SWI2*ΔABD deletion variants. Using the single-copy plasmid pECW24 (*ARS CEN URA3 SWI2*) and *SWI2*-specific primers, ΔABD variants lacking aa 240–298 and aa 160–298, respectively, were obtained by inverse PCR. The resulting plasmids pECW28 and pECW30 together with pECW24 as a positive control were transformed into a *swi2*Δ mutant and tested for complementation of mutant phenotypes (growth in the absence of inositol, utilization of carbon sources raffinose, ethanol and galactose, respectively). As is shown in Fig. 2 B, both *SWI2*ΔABD variants could fully restore pleiotropic growth deficiencies of the *swi2*Δ mutant, indicating that the ABD of Swi2 is functionally dispensable, presumably because other subunits of the SWI/SNF complex are able to compensate for its loss.

### SWI/SNF subunits Swi1, Snf5 and Snf6 also interact with Ino2

**Fig. 3:**
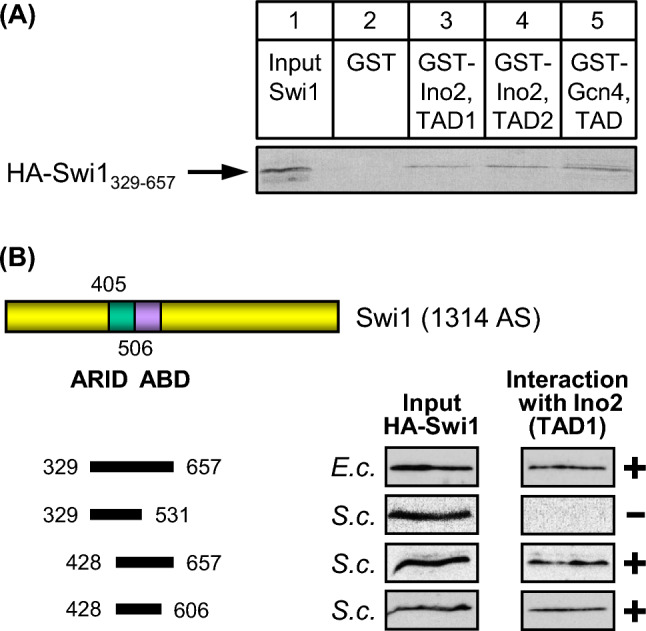
Swi1 interacts with TAD1 and TAD2 of Ino2. **A** HA-tagged Swi1 length variant aa 329–657 was synthesized in *S. cerevisiae* (expression plasmid pECW39) and assayed for binding to GST fusions with Ino2 TAD1 (pSH117), Ino2 TAD2 (pSH118) and Gcn4 TAD (pKH60; positive control). **B** Swi1 truncations were assayed for binding to GST-Ino2 TAD1 (pSH117). Expression plasmids pJuLu1 (aa 329–657), pMaS5 (aa 329–531), pMaS7 (aa 428–657) and pLM3 (aa 428–606) were used for synthesis of Swi1 variants in *E. coli* (*E.c.*) or in *S. cerevisiae* (*S.c.*). ABD, activator binding domain; ARID, AT-rich interaction domain

It was previously shown that the Gcn4 TAD not only interacts with Swi2 but also with SWI/SNF subunits Swi1 and Snf5 (Neely et al. 2002). Swi1 contains a mostly α-helical ARID (AT-rich interaction domain, aa 405–506) being able to bind DNA non-specifically (Wang et al. 2012). The ARID is contained within an internal sequence of Swi1 (aa 329–657) for which Prochasson et al. (2003) demonstrated interaction with activation domains of Gcn4 and VP16. We thus examined whether this region can also bind to activation domains of Ino2. Using Gcn4 TAD as a positive control we could show that both TADs of Ino2 indeed interact with epitope-tagged Swi1 in vitro (aa 329–657; Fig. 3A; lanes 3–5). For a more precise mapping of the ABD, truncated length variants of Swi1 were constructed and assayed for binding to Ino2 TAD1. As is shown in Fig. 3B, Swi1 variant aa 428–606 was still able to interact with TAD1 efficiently while variant aa 329–531 (comprising the entire ARID) failed to bind. Assuming that the DNA-binding ARID and the Swi1 ABD do not functionally overlap, aa 507–606 may represent the essential core of the ABD. Mapping studies with Ino2 TAD2 gave identical results (not shown).

**Fig. 4:**
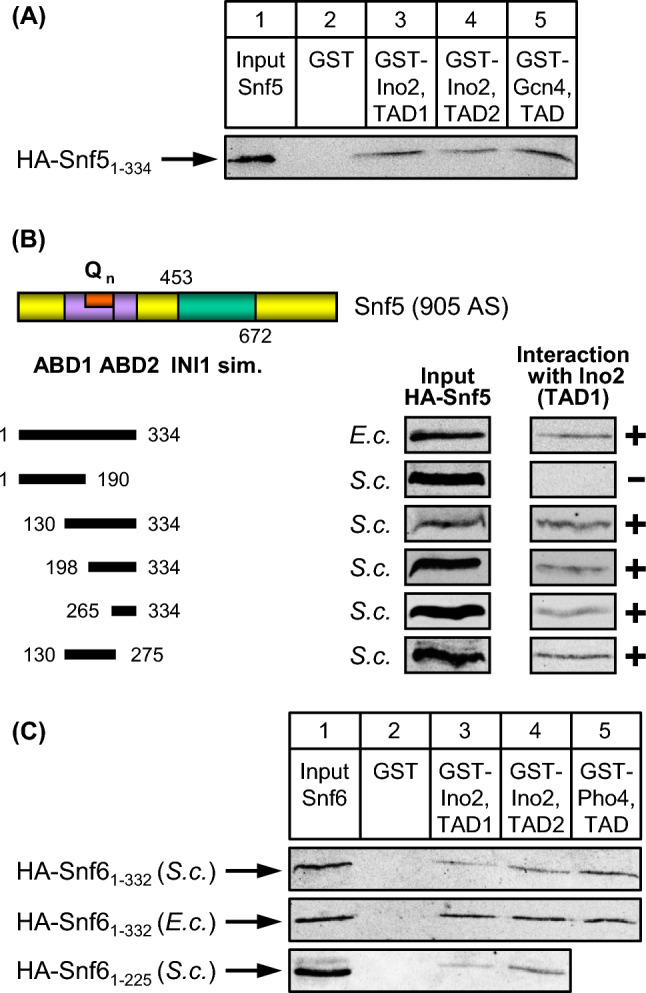
Snf5 and Snf6 interact with TAD1 and TAD2 of Ino2. **A** HA-tagged Snf5 length variant aa 1–334 was synthesized in *S. cerevisiae* (expression plasmid pECW40) and assayed for binding to GST fusions with Ino2 TAD1 (pSH117), Ino2 TAD2 (pSH118) and Gcn4 TAD (pKH60; positive control). **B** Snf5 truncations were assayed for binding to GST-Ino2 TAD1 (pSH117). Expression plasmids pJuLu2 (aa 1–334), pMaS6 (aa 1–190), pECW49 (aa 130–334), pECW50 (aa 198–334), pLM1 (aa 265–334) and pLM2 (aa 130–275) were used to synthesize Snf5 variants in *E. coli* (*E.c.*) or in *S. cerevisiae* (*S.c.*). ABD, activator binding domains; Q_n_, Glutamine-rich region; INI1 sim., region similar to human tumor suppressor INI1. **C** Snf6 interacts with activators Ino2 and Pho4. Full-length HA-Snf6 was synthesized in *S. cerevisiae* and in *E. coli* (expression plasmids pKB1 and pKB5, respectively) and assayed for binding to GST fusions with Ino2 TAD1 (pSH117), Ino2 TAD2 (pSH118) and Pho4 TAD (aa 1–104, pRAR62; positive control). Snf6 truncation variant aa 1–225 (pECW65) was synthesized in *S. cerevisiae*

Snf5 contains an internal sequence similar to the human INI1 protein (integrase interactor 1; aa 453–672, depicted in Fig. 4B) which was initially identified as a binding partner of HIV integrase and later shown to function as a tumor suppressor (Kalpana et al. 1994). The same domain of Snf5 is also similar to the Sfh1 subunit of chromatin remodelling complex RSC (“Snf Five Homolog”). The N-terminal sequence of Snf5 (aa 1–334) was previously shown to interact with VP16 and Gcn4 activation domains (Prochasson et al. 2003). This sequence contains a glutamine-rich region of unknown function (aa 221–270: 94% Gln; Q_n_ in Fig. 4B). As is apparent from Fig. 4A, aa 1–334 of Snf5 can be also bound by both TADs of Ino2 (lanes 3 and 4; TAD of Gcn4 as a positive control, lane 5). The same result was obtained with bacterially produced Snf5 aa 1–334 (Fig. 4B). We next synthesized shorter length variants of Snf5 and assayed for interaction with TAD1 of Ino2. The results of these studies argue for the existence of two activator binding domains within Snf5, ABD1 (aa 130–275) and ABD2 (aa 265–334; Fig. 4B). Although these domains have a short overlap (11 amino acids, 7 of which are glutamine), this region is certainly too small to form the core of an ABD, arguing for individual interaction of ABD1 and ABD2 with TADs.

Snf6 is an additional subunit of SWI/SNF which has been described as an interaction partner of activators Pho4 and Swi5 (Neely et al. 2002) but is devoid of sequences similar to known functional domains. We could demonstrate that TAD1 and TAD2 of Ino2 are also able to recruit Snf6 synthesized in yeast and in *E. coli* (Fig. 4C, lanes 3 and 4; using Pho4 as a positive control, lane 5), arguing for a direct interaction. This interaction may be mediated by Snf6 aa 1–225 which similarly bind to Ino2 TAD1 and TAD2. However, we cannot rule out the existence of an additional ABD since aa 1–225 was the only truncated Snf6 variant investigated.

SWI/SNF subunit Swi3 has been also demonstrated to bind selected activator proteins such as Swi5 (Neely et al. 2002). We thus finally investigated whether epitope-tagged Swi3 (full-length protein) can interact with TADs of Ino2, using Swi5 as a positive control. However, these experiments gave no evidence for Swi3 as an interaction partner of Ino2 (not shown).

### Mutational analysis of Sth1 interaction with transcriptional activation domains

**Fig. 5:**
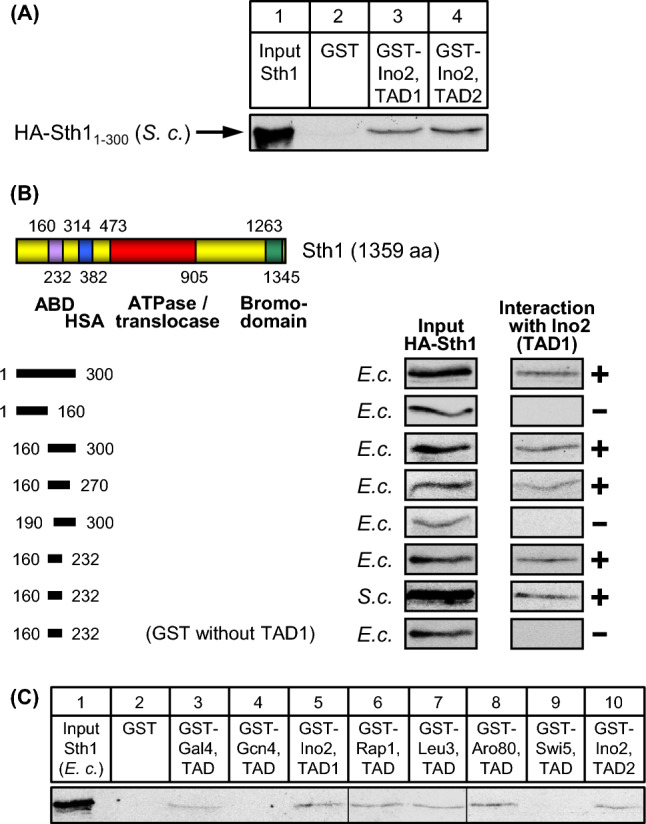
In vitro interaction assays (GST pull-down) with length variants of Sth1 and various activation domains. **A** Sth1 interacts with both Ino2 activation domains. GST fusion proteins containing TAD1 or TAD2 of Ino2 were synthesized in *E. coli*, immobilized on GSH sepharose and incubated with yeast protein extracts containing epitope-tagged Sth1 (aa 1–300; yeast expression plasmid pECW31). **B** Mapping of Sth1 domains interacting with TAD1 of Ino2. Expression plasmids pECW32 (aa 1–300), pECW34 (aa 1–160), pECW33 (aa 160–300), pMiM4 (aa 160–270), pMiM3 (aa 190–300) and pECW45 (aa 160–232) were used to synthesize HA-Sth1 variants in *E. coli* strain BL21 CodonPlus. Length variant aa 160–232 was also synthesized in *S. cerevisiae* (yeast expression plasmid pECW48). ABD, activator binding domain; HSA, helicase/SANT-associated domain. ATPase RecA subdomains are not depicted individually. Functional domain SnAC (aa 1001–1069) is omitted from the figure. **C** TADs of various unrelated transcriptional activators interact with Sth1. GST fusions of Ino2 TAD1, Ino2 TAD2, Gal4 TAD Gcn4 TAD, Leu3 TAD, Rap1 TAD, Aro80 TAD and Swi5 TAD were synthesized in *E. coli*, immobilized on GSH sepharose and incubated with bacterial protein extract containing epitope-tagged Sth1 length variant aa 1–300 (pECW32). Empty GST plasmid pGEX-2TK was used as a negative control

Molecular anatomy of ATPases Swi2 and Sth1 of chromatin remodelling complexes SWI/SNF and RSC, respectively, is highly conserved. We thus reasoned that Sth1 may possibly also interact with TADs of Ino2, mediated by a region at a similar position as demonstrated above for Swi2. A length variant of Sth1 (aa 1–300) could be stably synthesized in *S. cerevisiae* and indeed interacted with both TADs of Ino2 (Fig. 5A). Using various Sth1 truncations of its N-terminus, we finally identified a length variant of 73 amino acids (aa 160–232) as activator-binding domain which could be synthesized in *E. coli* and in yeast and was able to bind Ino2 TAD1, arguing for a direct interaction which is not dependent on other proteins from *S. cerevisiae* (Fig. 5B). The ABD of Sth1 is contained within the so-called “scaffold II”-domain (aa 154–318) which was identified by cryo-EM analysis of the RSC complex (Patel et al. 2019; scaffold I and II together are part of the “body module”; Wagner et al. 2020). As described above for Swi2, we also studied whether additional unrelated TADs can interact with Sth1. As shown in Fig. 5C, TADs of activators Gal4, Rap1, Leu3 and Aro80 were indeed able to bind the ABD of Sth1. It should be mentioned that we cannot exclude the existence of an additional ABD within the remaining sequence of Sth1 which was not investigated in this work.

**Fig. 6:**
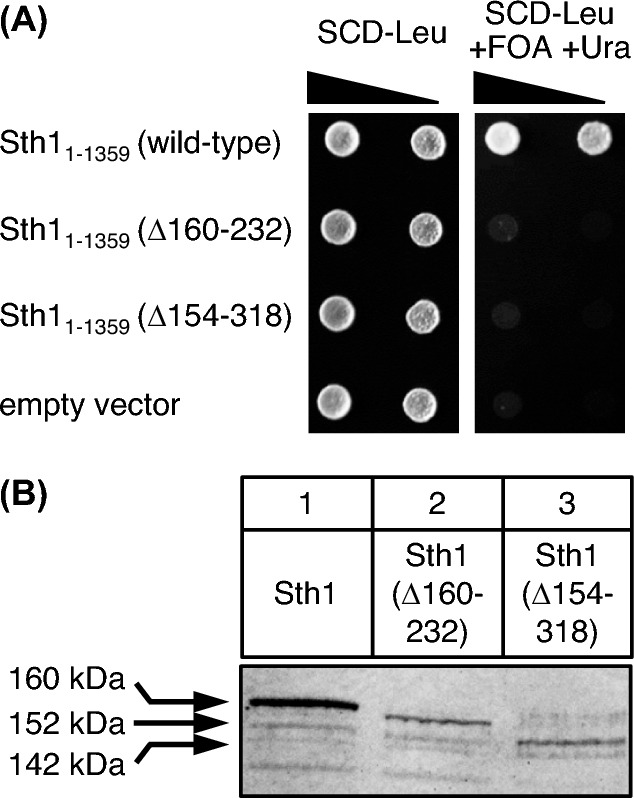
Assay for functional complementation of *sth1* mutation by *STH1* variants lacking ABD sequences. **A** *S. cerevisiae* strain KSY1 (relevant genotype: *ura3 leu2 sth1*Δ*::HIS3* + [pKS2: *ARS CEN URA3 STH1*]) was transformed with plasmids pKS4 (*ARS CEN LEU2 STH1*; wild-type), pKS7 (*STH1*Δ160-232), pKS8 (*STH1*Δ154-318) and YCplac111 (empty vector), respectively. Strain dilutions were spotted on SCD-Leu (one day) and SCD-Leu + FOA + Ura (counter-selection against rescue plasmid pKS2, two days). FOA, 5-fluoroorotic acid. **B** Detection of HA-Sth1 variants in strain C13-ABY.S86, transformed with expression plasmids pKS23 (*MET25-HA*_3_*-STH1*), pKS24 (*MET25-HA*_3_*-STH1*Δ160-232) and pKS25 (*MET25-HA*_3_*-STH1*Δ154-318), respectively. In all lanes, 50 µg of total protein were analyzed

To investigate whether a variant of the essential *STH1* gene lacking its ABD can functionally complement a *sth1* null mutation, we used the plasmid shuffle strategy, introducing a centromeric *URA3 STH1* rescue plasmid into a wild-type strain with subsequent deletion of the genomic *STH1* copy to give strain KSY1. Using a centromeric *LEU2 STH1* plasmid as a template, we next constructed two deletion variants by inverse PCR, lacking the core ABD (aa 160–232) and the more extended scaffold II-region (aa 154–318), respectively. Following transformation of these plasmids into strain KSY1, transformants were cultivated in the presence of FOA to select for loss of the *URA3 STH1* rescue plasmid. As is shown in Fig. 6A, neither *STH1*(Δ160–232) nor *STH1*(Δ154–318) could functionally replace *STH1*, indicating that the deleted domains are indispensable for the function of the protein. Truncated Sth1 variants as epitope-tagged proteins could be detected in cellular extracts (although at a reduced level; Fig. 6 B). We conclude that the failure to complement a *sth1* null mutation is not caused by a general instability of the resulting proteins.

**Fig. 7:**
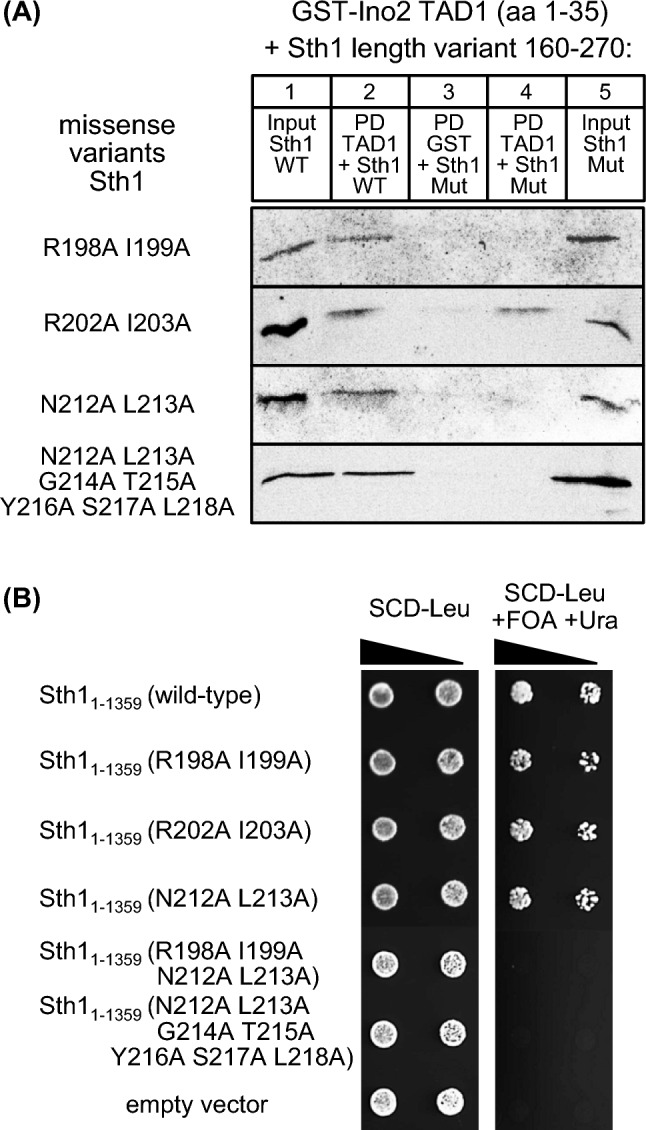
Functional analysis of missense variants introduced into the ABD of Sth1. **A** Comparative interaction studies of Ino2 TAD1 and variants of the Sth1 activator binding domain. Fusion protein GST-Ino2 (TAD1) was incubated with bacterial protein extracts containing epitope-tagged Sth1 (plasmid pMiM4, encoding wild-type aa 160–270) as well as variants R198A I199A (pLT3), R202A I203A (pLT2), N212A L213A (pLT4) and N212A L213A G214A T215A Y216A S217A L218A (pLT5). Input samples are shown in lanes 1 (wild-type, wild-type) and lanes 5 (mutant variant, Mut). Pull-down (PD) experiments were analyzed in lanes 2 (wild-type) and lanes 4 (mutant variant). **B** Plasmid shuffling experiments with *STH1* wild-type and missense variants encoding full-length proteins. *S. cerevisiae* strain KSY1 was transformed with plasmids pKS4 (*ARS CEN LEU2 STH1*; wild-type), pECW76 (R198A I199A), pECW77 (R202A I203A), pECW78 (N212A L213A), pECW79 (R198A I199A N212A L213A), pECW80 (N212A L213A G214A T215A Y216A S217A L218A) and YCplac111 (empty vector), respectively. Strain dilutions were spotted on SCD-Leu (one day) and SCD-Leu + FOA + Ura (counter-selection against rescue plasmid pKS2, two days). FOA, 5-fluoroorotic acid

Although we hypothesize that the essential regions deleted in *STH1*(Δ160–232) and *STH1*(Δ154–318) mediate recruitment of Sth1 to DNA-bound activators, it cannot be excluded that other functions such as formation of the RSC complex are also affected. We thus investigated activator binding of Sth1 more precisely by introduction of missense mutations at selected positions. Since evolutionary conservation of amino acids may indicate functional importance, we aligned sequences from various yeasts similar to the ABD of *S. cerevisiae* Sth1 (aa 160–232; Supplementary Fig. S3). As previously shown for TAF subunits of TFIID, combinations of basic and hydrophobic residues may be important for coactivator recruitment (Hintze et al. 2017). We thus replaced such amino acids by alanine (Sth1 R198A I199A, R202A I203A, N212A L213A and N212A L213A G214A T215A Y216A S217A L218A, respectively), synthesized mutational Sth1 variants in *E. coli* and used them for in vitro interaction studies with TAD1 of Ino2. It should be emphasized that the heptapeptide motif aa 212–218 is almost completely conserved among the yeast species compared (Fig. S3). While binding of variant R202A I203A was unaffected, variants R198A I199A and N212A L213A could no longer interact with Ino2 TAD1 in vitro (Fig. 7A). This result differs from what we have shown above for the ABD of Swi2 which had turned out as resistant to mutational modification. To study the influence of these mutations in vivo, we constructed corresponding variants of full-length *STH1* which were tested for functional complementation of a *sth1* null mutation by plasmid shuffling (Fig. 7B). To exclude severe alterations of Sth1 conformation by alanine replacements, we compared structural predictions for wild-type and mutant variants using AlphaFold (Jumper et al. 2021). No significant difference is predicted for variants R198A I199A, R202A I203A and N212A L213A while the septuple variant affecting aa 212–218 may exhibit an extended α-helix, possibly as a result of conversion of a conserved glycine into alanine (Supplementary Fig. S4). Although double variants R198A I199A and N212A L213A were functional in vivo, a combination of both of them (giving the quadruple variant R198A I199A N212A L213A) could no longer replace wild-type *STH1*. This was also true for the septuple variant N212A L213A G214A T215A Y216A S217A L218A which had failed to interact with Ino2 in vitro (Fig. 7A). In summary we conclude that the Sth1 sequence aa 198–218 represents the core of the protein being required for interaction with transcriptional activators.

### Identification of a multifunctional activator binding domain in ATPase Ino80

**Fig. 8:**
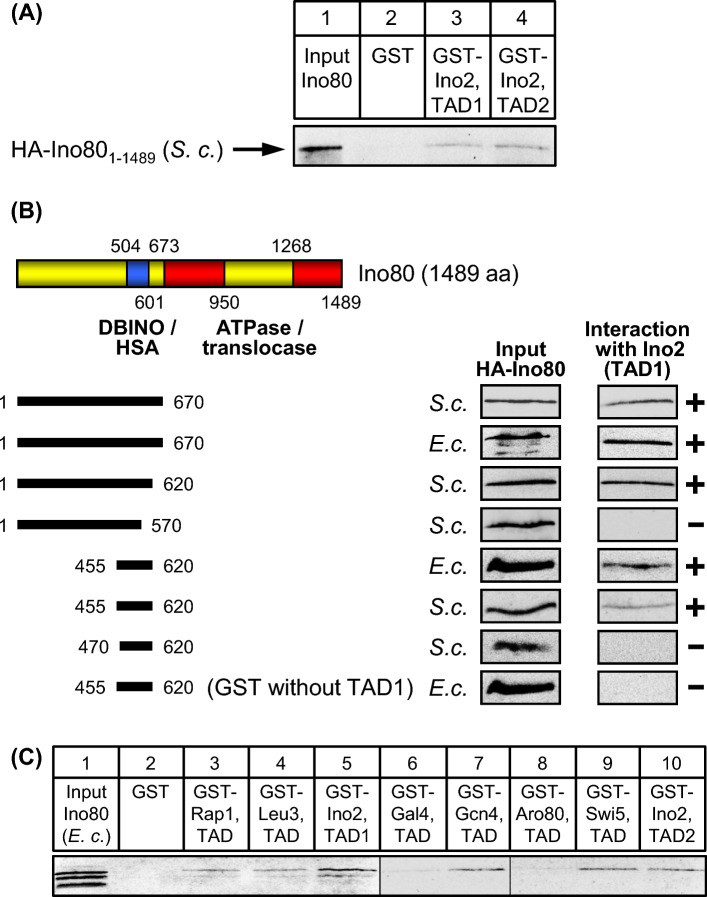
Ino80 interacts with various activation domains in vitro. **A** GST pull-down assays with Ino2 activation domains and full-length Ino80 synthesized in yeast (aa 1–1489, expression plasmid pRE82). **B** Mapping of Ino80 domains interacting with TAD1 of Ino2. Yeast and *E. coli* expression plasmids pECW38 (aa 1–670, S.c.), pMiM1 (aa 1–670, E.c.), pMM5 (aa 1–620, S.c.), pMM6 (aa 1–570, S.c.), pECW70 (aa 455–620, S.c.), pECW73 (aa 455–620, E.c.) and pECW72 (aa 470–620, S.c.) were used to synthesize HA-Ino80 length variants. DBINO, DNA-binding domain of Ino80. **C** TADs of various unrelated transcriptional activators interact with Ino80. GST fusions of Ino2 TAD1, Ino2 TAD2, Gal4 TAD Gcn4 TAD, Leu3 TAD, Rap1 TAD, Aro80 TAD and Swi5 TAD were synthesized in *E. coli*, immobilized on GSH sepharose and incubated with bacterial protein extract containing epitope-tagged Ino80 length variant aa 1–670 (pMiM1). Empty GST plasmid pGEX-2TK was used as a negative control

We have previously identified the pleiotropic *ino80* mutation leading to activation defects of genes involved in phospholipid biosynthesis and various unrelated metabolic pathways (Ebbert et al. 1999). The corresponding *INO80* gene encodes an ATPase (1489 aa) distantly related to Swi2 and Sth1 but similar to Swr1 (Bao and Shen 2007). The phenotype of *ino80* mutations indicates that Ino80 (or other subunits of the corresponding complex) may also interact with Ino2. Using an epitope-tagged variant of full-length Ino80, we were indeed able to demonstrate its binding to both TADs of Ino2 (Fig. 8A). For a more precise mapping of the domain mediating activator binding, we again used length variants of Ino80 and finally identified aa 455–620 as its ABD which was functional when synthesized either in yeast or in *E. coli* (Fig. 8B). Importantly, this sequence co-localizes with the previously identified DBINO (aa 504–601; Bakshi et al. 2004; Shen et al. 2003) and the HSA domain interacting with actin and actin-related proteins (aa 462–598; Szerlong et al. 2008). Not only TADs of Ino2 but also activators Rap1, Leu3, Gcn4 and Swi5 were able to interact with the ABD of Ino80 (Fig. 8C).

**Fig. 9:**
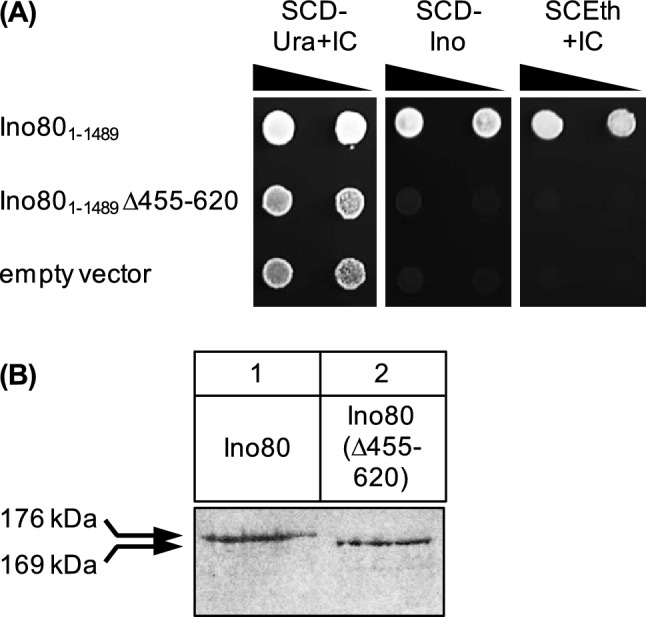
Assay for functional complementation of an *ino80* null mutation by *INO80* variant lacking the activator binding domain (ABD). **A** *S. cerevisiae* strain YAB1 (relevant genotype: *ura3 ino80*Δ*::kanMX*) was transformed with plasmids pRE49 (*ARS CEN URA3 INO80*; wild-type), pECW74 (*INO80*Δ455-620) and YCplac33 (empty vector), respectively. Serial dilutions of transformants were spotted on the media shown and incubated for one (SCD-Ura + IC), two (SCD-Ino) or three days (SCEth + IC). IC, inositol + choline; Ino, inositol; Eth, ethanol. **B** Detection of HA-Ino80 variant in strain C13-ABY.S86, transformed with expression plasmids pRE82 (*MET25-HA*_3_*-INO80*) and pECW75 (*MET25-HA*_3_*-INO80*Δ455-620), respectively. In all lanes, 50 µg of total protein were analyzed

Although an *INO80* gene deletion variant lacking sequences which encode amino acids 356–682 was unable to complement an *ino80* null mutation (Shen et al. 2003), we constructed an *INO80* variant devoid of its ABD as defined in this work (aa 455–620). The resulting protein could be stably synthesized in yeast (Fig. 9 B) but failed to complement an *ino80*Δ mutation (Fig. 9A), confirming the functional importance of Ino80 aa 455–620. Because of the versatile function of this domain, we cannot conclude that the observed deficiency of Ino80Δ455-620 is a result of missing activator recruitment. Similar to what we found for various subunits of SWI/SNF, other subunits of INO80 may be also contact partners of activator proteins.

## Discussion

In this work we investigated how transcriptional activators may contact CRC which then generate nucleosome-depleted regions in basal promoters, leading to improved access of general transcription factors and facilitating formation of the pre-initiation complex which stimulates the rate of RNA synthesis. Promoter recruitment of CRCs is mediated by their interaction with sequence-specific transcriptional activators but can be intensified by reading chromatin modifications such as acetyllysine bound by bromodomains which are present in ATPases Swi2 and Sth1 (but not in Ino80). Although nucleosome sliding is a common property of SWI/SNF, RSC and INO80 (reviewed by Eustermann et al. 2024), none of these complexes can compensate a single CRC deficiency and must therefore fulfill individual functions. While previous work has shown that several subunits of SWI/SNF can bind to activation domains (Neely et al. 1999, 2002; Prochasson et al. 2003), it remained unclear how activity of RSC and INO80 is stimulated by TADs. Initially concentrating on activator Ino2 of yeast phospholipid biosynthetic genes we showed that Ino2 can interact with four different subunits of SWI/SNF (Swi1, Swi2, Snf5 and Snf6) as well as with ATPases Sth1 and Ino80 of CRC RSC and INO80, respectively. Activator binding domains (ABDs) previously identified within Swi1 and Snf5 (Prochasson et al. 2003) could be further localized in this work. Although the domain organization of ATPase Swr1 is related to Ino80, we were unable to show binding of Ino2 to an epitope-tagged length variant of Swr1 (aa 1–470 comprising its HSA domain; results not shown). We extended our analyses beyond Ino2 and demonstrated that ATPases Swi2, Sth1 and Ino80 also contact a large number of functionally unrelated activators each of which displayed an individual pattern of TAD interactions (summarized in Table 1). Nevertheless, we cannot exclude that other subunits of RSC and INO80 are also able to bind TADs.

**Table 1:** Summary of interaction studies of various TADs and ATPase subunits of chromatin remodelling complexes SWI/SNF (Swi2), RSC (Sth1) and INO80 (Ino80)

Coactivator ATPase	Ino2 TAD1	Ino2 TAD2	Gal4 TAD	Gcn4 TAD	Rap1 TAD	Leu3 TAD	Aro80 TAD	Swi5 TAD
Swi2	+	+	+	+	+	-	+	+
Sth1	+	+	+	-	+	+	+	-
Ino80	+	+	-	+	+	+	-	+

Although we used Ino2 TAD1 for all mapping studies, minimal ABDs identified in Swi1, Swi2, Snf5, Sth1 and Ino80 could also bind to Ino2 TAD2 (results not shown). Comparison of these minimal ABDs did not show a conserved sequence pattern of specific amino acid residues. We could identify ABDs in Swi2 and Sth1 at similar positions both comprising ~ 70 amino acids, confirming a highly related molecular organization of functional domains in both ATPases. Interestingly, Swi2 devoid of its ABD was fully functional presumably because ABDs of Swi1, Snf5 and/or Snf6 can compensate for this loss. Previously, Prochasson et al. (2003) constructed gene variants of *SWI1* and *SNF5* lacking the coding region of the respective ABDs and observed that single deletions still allowed growth without inositol as we also found for *SWI2*(ΔABD). However, combined variants of Swi1 and Snf5 devoid of the respective ABDs led to a substantially reduced but not completely abolished growth of the strain in the absence of inositol. Results of this work indicate that ABDs of Swi2 and/or Snf6 may be responsible for the remaining growth observed. We conclude that various subunits of SWI/SNF can be redundantly recruited by activators. Consequently, loss of a single ABD must not result in a mutant phenotype.

Importantly, functional dynamics of SWI/SNF is controlled by post-translational protein modifications. Gcn5-dependent acetylation of Swi2 (at its AT-hook domain) impairs binding of SWI/SNF to promoter chromatin and facilitates its release from acetylated histones (Kim et al. 2010). Thus, ABD interactions with activation domains may be also influenced by post-translational modifications. However, our interaction experiments comparatively performed with protein extracts from yeast and *E. coli* gave identical results. We conclude that TAD-ABD interactions studied in this work do not require other yeast proteins or post-translational modifications specific for eukaryotes.

**Fig. 10:**
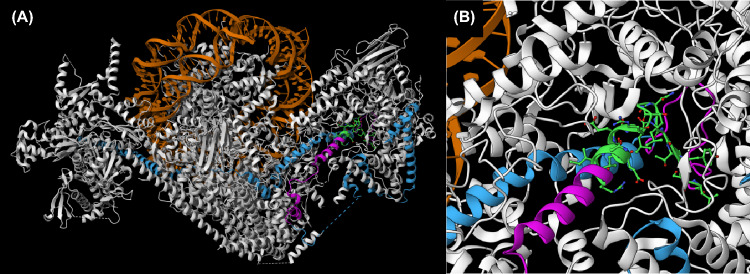
Structural model of RSC based on cryo-EM analysis of Patel et al. (2019). **A** View of entire RSC complex (PBD: 6V92) with functional and structural domains of Sth1 depicted in different colours: DNA of the nucleosome (orange), Sth1 (blue), ABD as defined by in vitro interaction experiments (aa 160–232, purple), core region of ABD as defined by mutational analysis (aa 200–218, green). **B** Enlarged display of ABD and its core region

In contrast to what we observed with Swi2(ΔABD), Sth1(ΔABD) could not replace the wild-type protein. This finding indicates that Sth1 possibly is the sole ABD-containing subunit of RSC or is at least of major importance for complex recruitment by activators. Indeed, Rsc9 and Sfh1 of RSC are substantially smaller than their ABD-containing SWI/SNF counterparts Swi1 and Snf5, respectively, and may do not contain ABDs. For both protein pairs, no similarity could be detected with the ABD-containing regions of Swi1 and Snf5. The similarity of Snf5 and Sfh1 is limited to a region of ~ 200 aa which is also similar to human tumor suppressor Ini1 (= SmarcB1, Baf47). Nevertheless, we could not exclude that formation of the RSC complex is also affected by deletion of the Sth1 ABD. We thus investigated the functional importance of Sth1 ABD more precisely and introduced missense mutations at evolutionary conserved basic and hydrophobic residues. Interaction assays showed that double mutations R198A I199A and N212A L213A can no longer bind to Ino2 TAD1 in vitro and structural comparisons predict that the conformation of both Sth1 variants should not be altered. Importantly, introduction of the quadruple mutation R198A I199A N212A L213A into full-length *STH1* created a variant which was unable to complement a *sth1* null mutation in vivo. Together, these results provide strong evidence that the existence of an ABD mediating recruitment by activators is indispensable for a functional Sth1 ATPase. The structure of RSC solved by cryo-EM (Patel et al. 2019) allowed us to conclude that the core ABD of Sth1 (aa 198–218) is positioned at the surface of the complex and thus should be accessible for activation domains (cf. Fig. 10). In contrast to what we found for Sth1, introduction of missense mutations at conserved residues into the ABD of Swi2 did not abolish its interaction with Ino2 TAD in vitro.

ABDs of Swi2 and Sth1 are located in the N-terminus of both proteins close to but separate from HSA domains required for recruitment of actin-related proteins (Arps). In contrast, our functional characterization of Ino80 revealed that its ABD physically overlaps with domains HSA and DBINO for which DNA-binding was initially predicted by bioinformatic analysis (Bakshi et al. 2004) and later demonstrated experimentally (Bakshi et al. 2006). More recent structural work demonstrated interaction of the Ino80 HSA domain with nuclear actin, Arp4 and Arp8 and its simultaneous role as a sensor for extranucleosomal linker DNA (Brahma et al. 2018; Knoll et al. 2018). This triple function as a HSA/DBINO/ABD may explain, why the ABD of Ino80 is substantially extended (aa 455–620) when compared with ABDs of Swi2 and Sth1 which comprise ~ 70 aa. Structural studies by Kunert et al. (2022) performed with Ino80 from the fungus *Chaetomium thermophilum* support the conclusion that this domain should still allow access for binding of activation domains, despite simultaneous interaction with actin, Arps and linker DNA. These authors also replaced a number of basic amino acids by alanine and observed strongly reduced DNA binding. Future work should show whether specific mutations introduced into the trifunctional HSA/DBINO/ABD may deactivate only a single function without affecting the remaining roles.

It was shown previously that CRCs SWI/SNF and INO80 are required for movement of nucleosomes at the repressed *INO1* promoter when derepression occurs (Ford et al. 2007). However, it is an open question whether CRCs are recruited to promoters only under conditions of gene induction or whether they are associated with promoters prior to occurrence of inducing conditions. Studying the *HO* promoter by chromatin immunoprecipitation experiments (ChIP), Cosma et al. (1999) showed cell cycle-regulated and Swi5-dependent recruitment of SWI/SNF. In contrast, Ford et al. (2008) described the presence of complexes SWI/SNF and INO80 at the *INO1* promoter under repressing and derepressing conditions. However, it should be mentioned that the experimental ChIP data of Ford et al. (2008) which were analyzed by quantitative real-time PCR are hardly above the threshold of significance. More recently, a comprehensive investigation of > 5000 yeast control regions provided strong evidence that pleiotropic cofactors such as SWI/SNF, SAGA and others are bound to regulated promoters prior to induction (Mittal et al. 2022). We conclude that interaction of activation domains with activator binding domains may fulfill a dual function, namely (I) recruitment of coactivators (here: chromatin remodelling complexes) to target promoters irrespective of the regulatory situation and (II) stimulation of coactivator activity (here: sliding of nucleosomes to create a nucleosome-depleted region) once a regulatory signal has been generated.

## Possible limitations of our work

This work demonstrates that 4 of the 12 subunits of complex SWI/SNF are able to interact with activator Ino2. We focused on the ABD of Swi2 which had not been characterized in previous work. Molecular analysis of ATPase subunits Sth1 and Ino80 of complexes RSC and INO80, respectively, showed that ABDs of both proteins are functionally indispensable. However, we cannot exclude that other subunits of RSC and INO80 not investigated in this work also contain ABDs. Since ATPase Swr1 of complex SWR1 is related to Ino80, we examined the N-terminus of Swr1 including its HSA domain for interaction with Ino2. Although no interaction was detected with this truncated protein, it remains open whether other sequences of Swr1 or distinct subunits of SWR1 contain ABDs.

We constructed mutational variants of Sth1 ABD and demonstrate that certain variants fail to interact with Ino2 TAD1 in vitro and are unable to complement a *sth1* null mutation in vivo. Since these modifications should create no or minimal local alterations of protein structure, Sth1 folding may not be affected, providing good evidence that the ABD of Sth1 is an essential domain. Despite this limited change we cannot completely rule out that mutations also affect formation of the RSC complex.

## Supplementary Information

Below is the link to the electronic supplementary material.

Supplementary file1 (PDF 329 KB)

## Data Availability

No datasets were generated or analysed during the current study. Original data are available upon request. Additional information is provided in the Supplementary Material.
