# Supplementary material for: Transcriptional activation domains interact with ATPase subunits of yeast chromatin remodelling complexes SWI/SNF, RSC and INO80
Source: Curr Genet. 2024 Sep 5;70(1):15. doi: 10.1007/s00294-024-01300-x (PMC11377671; doi:10.1007/s00294-024-01300-x)
Supplement: Supplementary file 1 — Supplementary file1 (PDF 329 KB) [file 294_2024_1300_MOESM1_ESM.pdf]

Supplementary Online Material Wendegatz et al.:

Table S1: Strains of *Saccharomyces cerevisiae*:

Strain	Genotype
C13-ABY.S86	*MAT*α *ura3 leu2 pra1 prb1 prc1 cps1*
EWY3	*MAT*α *ura3 leu2 trp1 his3 gal4*Δ*::kanMX SWI2*
EWY4	*MAT*α *ura3 leu2 trp1 his3 gal4*Δ*::kanMX swi2*Δ*::HIS3*
JS91.15-23	*MAT*α *ura3 leu2 trp1 his3*
JS94.8-12	*MAT*α *ura3 leu2 swi2*Δ*::HIS3*
KSY1	*MAT*α *ura3 leu2 his3 sth1*Δ*::HIS3* + [pKS2: *ARS CEN URA3 STH1*]
YAB1	*MAT*α *ura3 leu2 his3 trp1 ino80*Δ*::kanMX*

Table S2: Plasmids constructed and used:

Plasmid	Genotype
p416-MET25	*ARS CEN URA3 MET25*$_{Pr}$ (Mumberg et al. 1994)
p426-MET25HA	2µm *URA3 MET25*$_{Pr}$-HA$_3$ (Mumberg et al. 1994)
pDG1	tac$_{Pr/Op}$-*GST-SWI5*$_{1-85}$
pECW24	*ARS CEN URA3 MET25*$_{Pr}$-*SWI2*
pECW28	*ARS CEN URA3 MET25*$_{Pr}$-*SWI2*Δ240-298
pECW30	*ARS CEN URA3 MET25*$_{Pr}$-*SWI2*Δ160-298
pECW31	2µm *URA3 MET25*$_{Pr}$-HA$_3$-*STH1*$_{1-300}$
pECW32	tet$_{Pr/Op}$-HA$_3$-*STH1*$_{1-300}$
pECW33	tet$_{Pr/Op}$-HA$_3$-*STH1*$_{160-300}$
pECW34	tet$_{Pr/Op}$-HA$_3$-*STH1*$_{1-160}$
pECW38	2µm *URA3 MET25*$_{Pr}$-HA$_3$-*INO80*$_{1-670}$
pECW39	2µm *URA3 MET25*$_{Pr}$-HA$_3$-*SWI1*$_{329-657}$
pECW40	2µm *URA3 MET25*$_{Pr}$-HA$_3$-*SNF5*$_{1-334}$
pECW41	2µm *URA3 MET25*$_{Pr}$-HA$_3$-*SWI2*$_{1-300}$
pECW45	tet$_{Pr/Op}$-HA$_3$-*STH1*$_{160-232}$
pECW48	2µm *URA3 MET25*$_{Pr}$-HA$_3$-*SNF6*$_{160-232}$
pECW49	2µm *URA3 MET25*$_{Pr}$-HA$_3$-*SNF5*$_{130-334}$
pECW50	2µm *URA3 MET25*$_{Pr}$-HA$_3$-*SNF5*$_{198-334}$
pECW65	2µm *URA3 MET25*$_{Pr}$-HA$_3$-*SNF6*$_{1-225}$
pECW70	2µm *URA3 MET25*$_{Pr}$-HA$_3$-*INO80*$_{455-620}$
pECW72	2µm *URA3 MET25*$_{Pr}$-HA$_3$-*INO80*$_{470-620}$

pECW73	tet$_{Pr/Op}$-HA$_3$-*INO80*$_{455-620}$
pECW74	*ARS CEN URA3 INO80*Δ455-620
pECW75	2µm *URA3 MET25*$_{Pr}$-HA$_3$-*INO80*Δ455-620
pECW76	*ARS CEN LEU2 STH1* R198A I199A
pECW77	*ARS CEN LEU2 STH1* R202A I203A
pECW78	*ARS CEN LEU2 STH1* N212A L213A
pECW79	*ARS CEN LEU2 STH1* R198A I199A N212A L213A
pECW80	*ARS CEN LEU2 STH1* N212A I213A G214A T215A Y216A S217A L218A
pES5	tac$_{Pr/Op}$-*GST-LEU3*$_{841-886}$
pES17	2µm *URA3 MET25*$_{Pr}$-HA$_3$-*SWI2*$_{1-1703}$ (full-length)
pES20	tac$_{Pr/Op}$-*GST-GAL4*$_{768-881}$
pGBD-C1	2µm *TRP1 ADH1*$_{Prom}$-*GAL4*$_{DBD}$ (James et al. 1996)
pGEX-2TK	tac$_{Pr/Op}$-*GST*
pIB1	tet$_{Pr/Op}$-HA$_3$-*SWI2*$_{1-160}$
pIB2	tet$_{Pr/Op}$-HA$_3$-*SWI2*$_{1-307}$
pIB3	tet$_{Pr/Op}$-HA$_3$-*SWI2*$_{161-450}$
pIB4	tet$_{Pr/Op}$-HA$_3$-*SWI2*$_{161-307}$
pIB5	tet$_{Pr/Op}$-HA$_3$-*SWI2*$_{301-450}$
pIB9	tet$_{Pr/Op}$-HA$_3$-*SWI2*$_{206-307}$
pIB12	tet$_{Pr/Op}$-HA$_3$-*SWI2*$_{238-307}$
pJuLu1	tet$_{Pr/Op}$-HA$_3$-*SWI1*$_{329-657}$
pJuLu2	tet$_{Pr/Op}$-HA$_3$-*SNF5*$_{1-334}$
pKB1	2µm *URA3 MET25*$_{Pr}$-HA$_3$-*SNF6*$_{1-332}$
pKB2	2µm *URA3 MET25*$_{Pr}$-HA$_3$-*SWI3*$_{1-825}$
pKB5	tet$_{Pr/Op}$-HA$_3$-*SNF6*$_{1-332}$
pKH29	2µm *URA3 GAL1-lacZ*
pKH60	tac$_{Pr/Op}$-*GST-GCN4*$_{9-172}$
pKS2	*ARS CEN URA3 STH1*
pKS4	*ARS CEN LEU2 STH1*
pKS7	*ARS CEN LEU2 STH1*Δ160-232
pKS8	*ARS CEN LEU2 STH1*Δ154-318
pKS10	*sth1Δ::HIS3*
pKS23	2µm *URA3 MET25*$_{Pr}$-HA$_3$-*STH1*
pKS24	2µm *URA3 MET25*$_{Pr}$-HA$_3$-*STH1*Δ160-232
pKS25	2µm *URA3 MET25*$_{Pr}$-HA$_3$-*STH1*Δ154-318
pLJ6	tac$_{Pr/Op}$-*GST-RAP1*$_{630-671}$
pLM1	2µm *URA3 MET25*$_{Pr}$-HA$_3$-*SNF5*$_{265-334}$

pLM2	2µm *URA3 MET25*$_{Pr}$-HA$_3$-*SNF5*$_{130\text{-}275}$
pLM3	2µm *URA3 MET25*$_{Pr}$-HA$_3$-*SWI1*$_{428\text{-}606}$
pLT2	tet$_{Pr/Op}$-HA$_3$-*STH1*$_{160\text{-}270}$ R202A I203A
pLT3	tet$_{Pr/Op}$-HA$_3$-*STH1*$_{160\text{-}270}$ R198A I199A
pLT4	tet$_{Pr/Op}$-HA$_3$-*STH1*$_{160\text{-}270}$ N212A I213A
pLT5	tet$_{Pr/Op}$-HA$_3$-*STH1*$_{160\text{-}270}$ N212A I213A G214A T215A Y216A S217A L218A
pMaS5	2µm *URA3 MET25*$_{Pr}$-HA$_3$-*SWI1*$_{329\text{-}531}$
pMaS6	2µm *URA3 MET25*$_{Pr}$-HA$_3$-*SNF5*$_{1\text{-}190}$
pMaS7	2µm *URA3 MET25*$_{Pr}$-HA$_3$-*SWI1*$_{428\text{-}657}$
pMG50	tac$_{Pr/Op}$-*GST-ARO80*$_{846\text{-}950}$
pMG140	tet$_{Pr/Op}$-HA$_3$-*SWI2*$_{1\text{-}450}$
pMG142	tet$_{Pr/Op}$-HA$_3$-*SWI2*$_{1\text{-}550}$
pMiM1	tet$_{Pr/Op}$-HA$_3$-*INO80*$_{1\text{-}670}$
pMiM3	tet$_{Pr/Op}$-HA$_3$-*STH1*$_{190\text{-}300}$
pMiM4	tet$_{Pr/Op}$-HA$_3$-*STH1*$_{160\text{-}270}$
pMM5	2µm *URA3 MET25*$_{Pr}$-HA$_3$-*INO80*$_{1\text{-}620}$
pMM6	2µm *URA3 MET25*$_{Pr}$-HA$_3$-*INO80*$_{1\text{-}570}$
pRAR62	tac$_{Pr/Op}$-*GST-PHO4*$_{1\text{-}104}$
pRE49	*ARS CEN URA3 INO80*
pRE82	2µm *URA3 MET25*$_{Pr}$-HA$_3$-*INO80*
pSG14	2µm *TRP1 ADH1*$_{Prom}$-*GAL4*$_{DBD}$-*INO2*$_{1\text{-}35}$ (TAD1)
pSG16	2µm *TRP1 ADH1*$_{Prom}$-*GAL4*$_{DBD}$-*INO2*$_{101\text{-}135}$ (TAD2)
pSH117	tac$_{Pr/Op}$-*GST-INO2*$_{1\text{-}35}$ (TAD1)
pSH118	tac$_{Pr/Op}$-*GST-INO2*$_{101\text{-}135}$ (TAD2)
YCplac33	*ARS CEN URA3* (Gietz and Sugino 1988)
YCplac111	*ARS CEN LEU2* (Gietz and Sugino 1988)

Pr, promoter; Op, operator; All positions refer to the protein encoded by the gene given.

Table S3: Oligonucleotides used (PCR primers for construction of length variants for *SWI1*, *SWI2*, *SWI3*, *SNF5*, *SNF6*, *STH1* and *INO80*; site-directed mutagenesis of *SWI2* and *STH1*):

Name	Gene	Position	Sequence 5‘-3‘
Ino80_1F_BamHI	*INO80*	+1/+22	gact**ggatcc**ATGTCACTGGCAGTTCTACT
Ino80_1489F_HindIII	*INO80*	+4465/+4485	gact**aagctt**ATCGATACCGTCGACCTCGA
Ino80_455F_BamHI	*INO80*	+1362/+1382	gact**ggatcc**ATGAAAATGAATGCTAAGGA

Ino80_455R_PstI	*INO80*	+1363/+1343	gact**ctgcag**GCCGTAAGTGGGCAACCCG T
Ino80_455R_SpeI	*INO80*	+1363/+1343	gact**actagt**GCCGTAAGTGGGCAACCCGT
Ino80_470F_BamHI	*INO80*	+1407/+1427	gact**ggatcc**GACAACACCTATACAACTAT
Ino80_470R_HindIII	*INO80*	+1410/+1387	gact**aagctt**CTAGTCGTAATGCCTCTGGA TAG
Ino80_520F_BamHI	*INO80*	+1554/+1577	gact**ggatcc**AAGAACTTCAAACAAATAAA
Ino80_530F_BamHI	*INO80*	+1588/+1607	gact**ggatcc**ACGAGGGCCAGAAGAGGTA T
Ino80_560F_BamHI	*INO80*	+1678/+1697	gact**ggatcc**GAGGCTATGGAACAGGCAA A
Ino80_600R_HindIII	*INO80*	+1800/+1780	gact**aagctt**CTATTTGATTTTCCTTCCAAT GAA
Ino80_620F_PstI	*INO80*	+1860/+1880	gact**ctgcag**GACATTTCAGCTTTGGCTCC
Ino80_620F_SpeI	*INO80*	+1860/+1880	gact**actagt**GACATTTCAGCTTTGGCTCC
Ino80_670R_HindIII	*INO80*	+2010/+1987	gact**aagctt**CTAATGATCATCAAATTGTTT CG
Snf5_1F_BamHI	*SNF5*	+1/+23	gcat**ggatcc**ATGAATAATCAGCCGCAGG GTAC
Snf5_334R_XhoI	*SNF5*	+1002/+979	gcat**ctcgag**CTATCCGTATCTGCTTTTTTG TC
Snf5_130F_BamHI	*SNF5*	+388/+409	gatc**ggatcc**TTACCTTTGGCTACACAGCA
Snf5_130R_XhoI	*SNF5*	+390/+367	gcat**ctcgag**CTACTGAGCAATTTGTGGAG GCA
Snf5_190F_BamHI	*SNF5*	+568/+588	gcat**ggatcc**GTTCAGCAGCAACAGCAGC A
Snf5_190R_XhoI	*SNF5*	+570/+547	gcat**ctcgag**CTACAGCAATTGCCTCTGCT GTT
Snf5_198F_BamHI	*SNF5*	+592/+613	gatc**ggatcc**CTTAGAAACCAAATACAGCG
Snf5_265F_SpeI	*SNF5*	+796/+815	gact**actagt**CAGCAGCAAGGACAAATACC
Snf5_275R_SalI	*SNF5*	+830/+809	gact**gtcgac**TTGCTGAGATTGCGGTATTT
Snf6_1F_*Bam*HI	*SNF6*	+1/+20	gcat**ggatcc**ATGGGTGTCATCAAGAAGAA
Snf6_225R_XhoI	*SNF6*	+675/+651	gcat**ctcgag**CTACGCCGGCGCTTCGCGG AGATC
Snf6_332R_*Xho*I	*SNF6*	+999/+976	gcat**ctcgag**TTAAAAAAATACAGCATCAA GATC
Sth1-Start-BamHI	*STH1*	+1/+21	gact**ggatcc**AAAATGCTTCAGGAACAATC TGAG
Sth1-Stop-HindIII	*STH1*	+881/+900	gact**aagctt**CTAGGTTTGTGGGACAATAA CTT
Sth1_160F_BamHI	*STH1*	+478/+498	gact**ggatcc**ATATCAGCAGATTTCAATGC
Sth1_160R_HindIII	*STH1*	+480/+460	gact**aagctt**GCATTGAAATCTGCTGATAT

Sth1_190F_BamHI	*STH1*	+568/+589	gact**ggatcc**GATACCGAAATAAGGATTTC
Sth1_232R_HindIII	*STH1*	+696/+673	gact**aagctt**CTAGGACGATAAATCATCTTTTG
Sth1_270R_HindIII	*STH1*	+810/+787	gcat**aagctt**CTAGTGTGCCTGGCTGGCAACAT
Sth1 154R XhoI	*STH1*	+462/+441	gact**ctcgag**AGAATAGTCTACTTCGATCGC
Sth1 160R XhoI	*STH1*	+480/+460	gatc**ctcgag**TTTTATTGGTTTCTTTTCAG
Sth1 318F XhoI	*STH1*	+952/+972	gatc**ctcgag**AAGGAACGTAATTTACATTT
Sth1 323F XhoI	*STH1*	+967/+988	gatc**ctcgag**AGAATGGATACTTTCAAAAT
Sth1_Prom_EcoRI	*STH1*	-295/-274	gact**gaattc**CCACAAAACAATATTTCCACAG
Sth1_Term_SalI	*STH1*	+4274/+4254	gact**gtcgac**GCGGCACTAATAAGCCCAGAG
Sth1_1970_KpnI	*STH1*	+1969/+1992	GATTGGTTTAATACTCCATTTGCC
Sth1_2040_KpnI	*STH1*	+2040/+2017	CAGAGTTTCTTCTTCCGTTAATTC
Sth1_3280_XbaI	*STH1*	+3274/+3296	gact**tctaga**AGGAGATTACGTCAAAATGG
Sth1_710_SacI	*STH1*	+711/692	gact**gagctc**GAAAGTATCCATTCTGGACG
Sth1 aa 160-270 R198A I199A 3R	*STH1*	+613/+565	CGTTAATTCTGTTGGATGCAGCTGCAGAAATCCTTATTTCGGTATCTGG
Sth1 aa 160-270 R198A I199A 5F	*STH1*	+565/+613	CCAGATACCGAAATAAGGATTTCTGCAGCTGCATCCAACAGAATTAACG
Sth1 aa 160-270 R202A I203A 3R	*STH1*	+630/+580	AGGTAATCTTTCCAGCTCGTTAGCTGCGTTGGATATACGTGCAGAAATCCT
Sth1 aa 160-270 R202A I203A 5F	*STH1*	+580/+630	AGGATTTCTGCACGTATATCCAACGCAGCTAACGAGCTGGAAAGATTACCT
Sth1 aa 160-270 N212A L213A_3R	*STH1*	+660/+613	ATCATCTAGTGAGTATGTACCCGCAGCGGCAGGTAATCTTTCCAGCTC
Sth1 aa 160-270 N212A L213A_5F	*STH1*	+613/+660	GAGCTGGAAAGATTACCTGCCGCTGCGGGTACATACTCACTAGATGAT
Sth1 aa 212-218 NLGTYSL_F	*STH1*	+618/+677	GGAAAGATTACCTGCCGCTGCGGCTGCAGCCGCAGCAGATGATTGTTTAGAGTTTATTAC
Sth1 aa 212-218 NLGTYSL_R	*STH1*	+677/+618	GTAATAAACTCTAAACAATCATCTGCTGCGGCTGCAGCCGCAGCGGCAGGTAATCTTTCC
Swi1_329F_BamHI	*SWI1*	+985/+1008	gcat**ggatcc**ATGAGAGCTGCAATGTTTGCCGC
Swi1_657R_XhoI	*SWI1*	+1971/+1948	gcat**ctcgag**CTACTTGTAGACTTTAGGCAATT
Swi1_428F_BamHI	*SWI1*	+1282/+1303	gcat**ggatcc**CAATCAATACCAGAAATCGG

Swi1_531R_XhoI	*SWI1*	+1596/+1572	gact**ctcgag**CTAGTTGGCATTGGCCAATGCAGC
Swi1_606R_SalI	*SWI1*	+1819/+1795	gac**tgtcgac**CTTTTTGGTCTTTTTTTTCACTCT
Swi2_BamHI_Start_1	*SWI2*	+1/+20	gatc**ggatcc**ATGAACATACCACAGCGTCA
Swi2_SalI_Stop_1703	*SWI2*	+5109/+5088	gatc**gtcgac**CTATACACTCGCTTCTGTCATG
Swi2_Stop_450_HindIII	*SWI2*	+1350/+1329	gatc**aagctt***TCA*AACCATCATATCAGGATCATCT
Swi2_Stop_550_HindIII	*SWI2*	+1650/+1630	gatc**aagctt***TCA*TAATACATGACCTCTTACGGC
Swi2_aa307_HindIII_3R	*SWI2*	+921/+905	gatc**aagctt**TCATGTGGGTTGTCTTCTTC
Swi2_aa161_BamHI_5F	*SWI2*	+481/+500	gatc**ggatcc**GAGAAACCAGATAACTCAAACC
Swi2_aa160_HindIII_3R	*SWI2*	+480/+462	gatc**aagctt**TCACGCAGCATTTTCTCCATTG
Swi2_aa206_BamHI_5F	*SWI2*	+616/+637	gatc**ggatcc**CCCCAGCAGGCGCAGATGCAGC
Swi2_aa238_BamHI_5F	*SWI2*	+712/+731	gatc**ggatcc**CAACAGGGGAGAAGATTACC
Swi2_aa301_BamHI_5F	*SWI2*	+901/+920	gatc**ggatcc**GCGAGAAGAAGACAACCCAC
Swi2-160R-BamHI	*SWI2*	+480/+460	gact**ggatcc**CGCAGCATTTTCTCCATTGT
Swi2_240R_BamHI	*SWI2*	+720/+700	gact**ggatcc**CTGTTGCAGTCTCGCTTGTG
Swi2_298F_BamHI	*SWI2*	+892/+912	gact**ggatcc**GAGTTTGCGAGAAGAAGACA
Swi2 aa 238-307 E251A-S253A 5F	*SWI2*	+734/+775	GACAATGTTTACTGCCGCGGCAGCCGAACTGTTAAAGGCCC
Swi2 aa 238-307 E251A-S253A 3R	*SWI2*	+775/+734	GGGCCTTTAACAGTTCGGCTGCCGCGGCAGTAAACATTGTC
Swi2 aa238-307 F248A T249A 5F	*SWI2*	+724/+763	GATTACCCATGACAATGGCTGCTGCCGAGCAATCCGAAC
Swi2 aa238-307 F248A T249A 3R	*SWI2*	+763/+724	GTTCGGATTGCTCGGCAGCAGCCATTGTCATGGGTAATC
Swi2 aa238-307 L256A K257A L263A K264A 5F	*SWI2*	+753/+806	GCAATCCGAACTGGCAGCGGCCCAAATCACATCTGCAGCATGTCTAGTAAATAG
Swi2 aa238-307 L256A K257A L263A K264A 3R	*SWI2*	+806/+753	CTATTTACTAGACATGCTGCAGATGTGATTTGGGCCGCTGCCAGTTCGGATTGC
Swi2 aa238-307 I260A T261A 5F	*SWI2*	+763/+799	CTGTTAAAGGCCCAAGCCGCATCTCTAAAATGTCTAG

Swi2 aa238-307<br>I260A T261A 3R	*SWI2*	+799/+763	CTAGACATTTTAGAGATGCGGC<br>TTGGGCCTTTAACAG
Swi2 aa238-307<br>L266A V267A N268A 5F	*SWI2*	+780/+823	CACATCTCTAAAATGTGCAGCAGCT<br>AGAAAGCCTATTCCGTTTG
Swi2 aa238-307<br>L266A V267A N268A 3R	*SWI2*	+823/+780	CAAACGGAATAGGCTTTCTAGCTGC<br>TGCACATTTTAGAGATGTG
Swi2 aa238-307<br>K270A P271A 5F	*SWI2*	+794/+841	GTCTAGTAAATAGAGCGGCTATTC<br>CGTTTGAATTTCAGGCTGTTATCC
Swi2 aa238-307<br>K270A P271A 3R	*SWI2*	+841/+794	GGATAACAGCCTGAAATTCAAAC<br>GGAATAGCCGCTCTATTTACTAGAC
Swi2 aa238-307<br>V279A I280A Q281A 5F	*SWI2*	+817/+857	CCGTTTGAATTTCAGGCTGCTGCCGCA<br>AAATCTATTAACC
Swi2 aa238-307<br>V279A I280A Q281A 3R	*SWI2*	+857/+817	GGTTAATAGATTTTGCGGCAGC<br>AGCCTGAAATTCAAACGG
Swi2 aa238-307<br>H286A P287A F290A K291A 5F	*SWI2*	+840/+891	CCAAAAATCTATTAACGCTGCTCCAG<br>ATGCCGCAAGAATGTTACTGTCTTTG
Swi2 aa238-307<br>H286A P287A F290A K291A 3R	*SWI2*	+891/+840	CAAAGACAGTAACATTCTTGCGGCAT<br>CTGGAGCAGCGTTAATAGATTTTTGG
Swi2 aa238-307<br>R292A M293A 5F	*SWI2*	+855/+903	CCATCCTCCAGATTTCAAAGCAGCG<br>TTACTGTCTTTGAGTGAGTTTGCG
Swi2 aa238-307<br>R292A M293A 3R	*SWI2*	+903/+855	CGCAAACTCACTCAAAGACAGTAA<br>CGCTGCTTTGAAATCTGGAGGATGG
Swi3_1F_*Bam*HI	*SWI3*	+1/+20	gcat**ggatcc**ATGGAGAATACACTGGGTGA
Swi3_825R_*Xho*I	*SWI3*	+2478/+2459	gcat**ctcgag**TTATGCCGACCAATACCTGT

Artificially inserted cleavage sequences for restriction enzymes are shown in **bold**; capital letters represent genuine gene-specific sequences; capital letters underlined indicate sequences introducing site-specific mutations (GCN for alanine).

Supplementary Figures:

```
                    ## ###  ##  ## ## ### ##       ###    ##  ####
Scer  238 QQGRRLPMTMFTAEQSELLKAQITSLKCLVNRKPIPFEFQAVIQKSINHPPDFKRM 293
Sarb  245 QQGKRLPMTMFTAEQSELLKAQITSLKCLVNRKPIPLEFQAVIQKSINHPPDFKRM 300
Cgla  215 QQRPVPQNSIFTPQQSELLRAQISALKSLVNNQPVPQEYQKVIQQSINNPPDFKRM 270
Agos   94 PAGGSGGHGTFTMEQSELLKAQIAALKCLAHKQPIPKEVLEVIQLSLNNPPNLRQM 149
Klact 153 NNNASSSRSLFTDEQSLLLKAQIQSLKMMANHMVVPPEIMMVIDRSLTNLLDFKSI 208
Cons.                FT  QS LLKAQI  LK +     +P E+  VI  S+     ++ +

Scer  294 LLSLSEFARRRQPT 307
Sarb  301 LLSLSEFAKRRQPI 314
Cgla  271 LLSLSDFVKKKQLN 284
Agos  150 VTMVSSVLAQRSAS 163
Klact 209 LLALSADLQAHNAG 222
Cons.     +  +S     +
```

Supplementary Fig. S1: Comparison of activator binding domain (ABD) sequences of proteins similar to Swi2 from various yeasts. Scer, *Saccharomyces cerevisiae*; Sarb, *Saccharomyces arboricola*; Cgla, *Candida glabrata*; Agos, *Ashbya gossypii*; Klac, *Kluyveromyces lactis*. Identical amino acids are indicated by grey shadowing; mutagenized residues within *S. cerevisiae* Swi2 are indicated by #.

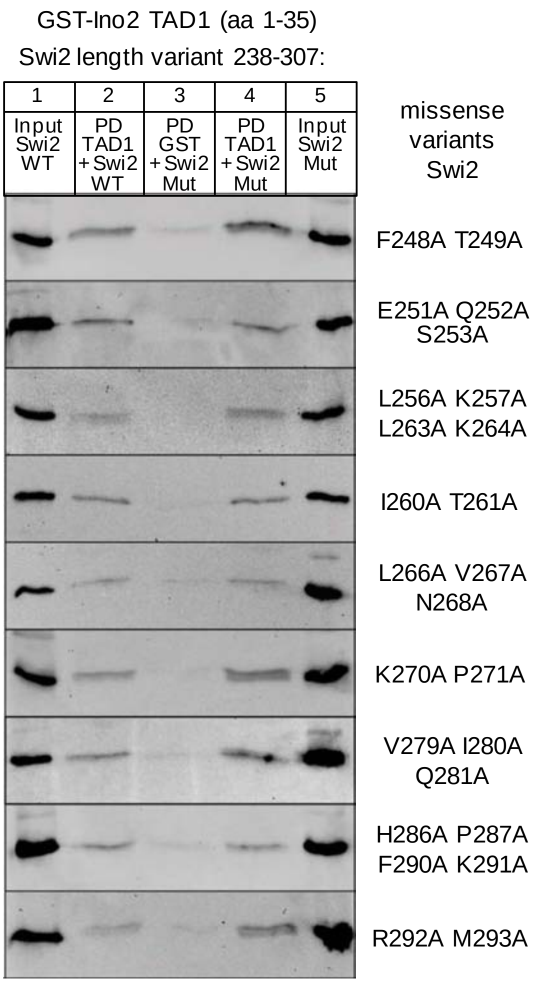

Supplementary Fig. S2: Comparative studies of interaction between Ino2 TAD1 and variants of Swi2 activator binding domain. GST fusion protein representing TAD1 of Ino2 (pSH117) was incubated with bacterial protein extracts containing epitope-tagged Swi2 (plasmid pIB12, encoding wild-type aa 238-307) as well as variants F248A T249A (pECW1), E251A Q252A S253A (pECW2), L256A K257A L263A K264A (pMS172), I260A T261A (pECW3), L266A V267A N268A (pECW4), K270A P271A (pMS173), V279A I280A Q281A (pECW5), H286A P287A F290A K291A (pMS174) and R292A M293A (pMS175). Input samples are shown in lanes 1 (wild-type, WT) and lanes 5 (mutant variant, Mut). Pull-down (PD) experiments were analyzed in lanes 2 (wild-type) and lanes 4 (mutant variant).

```
Scer   160 KISADFNAKAKSLGLESKFSN-----ATKTALGDPDTEI 193
Skud   160 KISMDFNAKAKSLGLESKFSN-----TSKTALGDPETEI 193
Cgla   142 KLAYNLDENAELLGMKDSFTL----VTLKNQLGNRETEE 176
Agos   143 QLSLDFTANAEKFGLKDKFCS-IGYQKTSSKLGEYKMET 180
Klac   199 KLSVDFDETSVRLGLPEALSNFRLKSGNEASLGDWEVEK 237
Cons.        +  +      +G+   +            LG    E

               ##  ##        #######
Scer   194 RISARISNRINELERLPANLGTYSLDDCLEFITKDDLSS 232
Skud   194 RISARISNRISELERLPANLGTYSLDDCLEFITKDDLSS 232
Cgla   177 IISTRIAKRIFELERLPGNLATYSLNDCLDFISKNDAPS 215
Agos   181 AISARIAQRIHELETLPSNLGTYSLDDALEFITKGDVPS 219
Klac   238 IISTLIAKRIKQLENLPSNLGTYSLNDALDFVTKDDIPT 276
Cons.       IS  I  RI  LE LP NL+TYSL D L+F++K D+
```

Supplementary Fig. S3: Comparison of activator binding domain (ABD) sequences of proteins similar to Sth1 from various yeasts. Scer, *Saccharomyces cerevisiae*; Sarb, *Saccharomyces arboricola*; Cgla, *Candida glabrata*; Agos, *Ashbya gossypii*; Klac, *Kluyveromyces lactis*. Identical amino acids are indicated by grey shadowing; mutagenized residues within *S. cerevisiae* Sth1 are indicated by #.

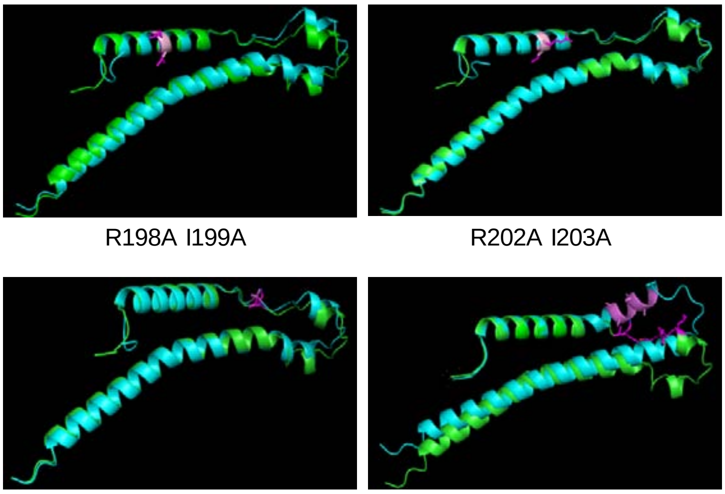

R198A I199A

R202A I203A

N212A L213A

N212A L213A G214A T215A
Y216A S217A L218A

Supplementary Fig. S4: Comparative structural prediction of Sth1 ABD wild-type and mutant variants.

(A)

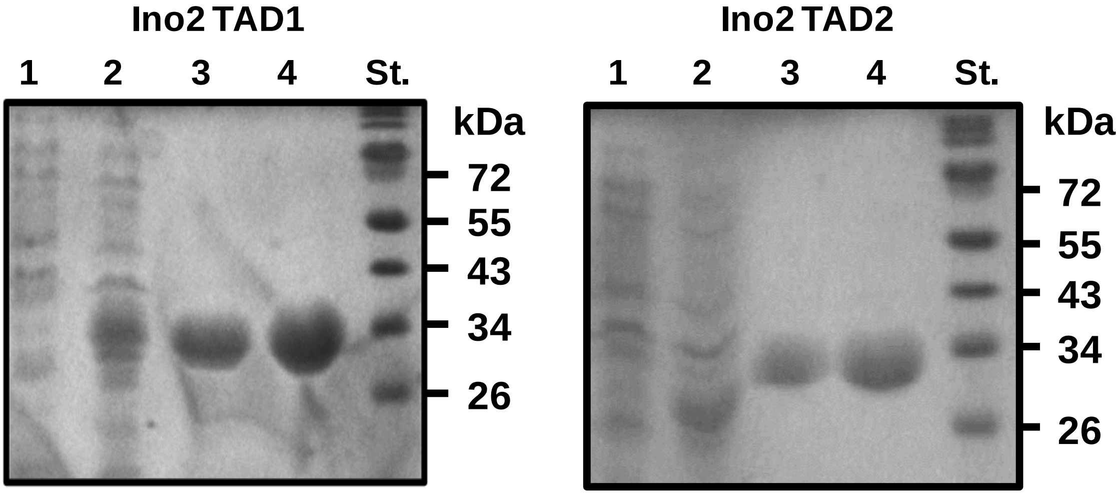

1 Protein extract, non-induced culture
2 Protein extract, induced with IPTG
3 Eluate from GSH sepharose
4 GSH sepharose-bound protein
St. Size standard

(B)

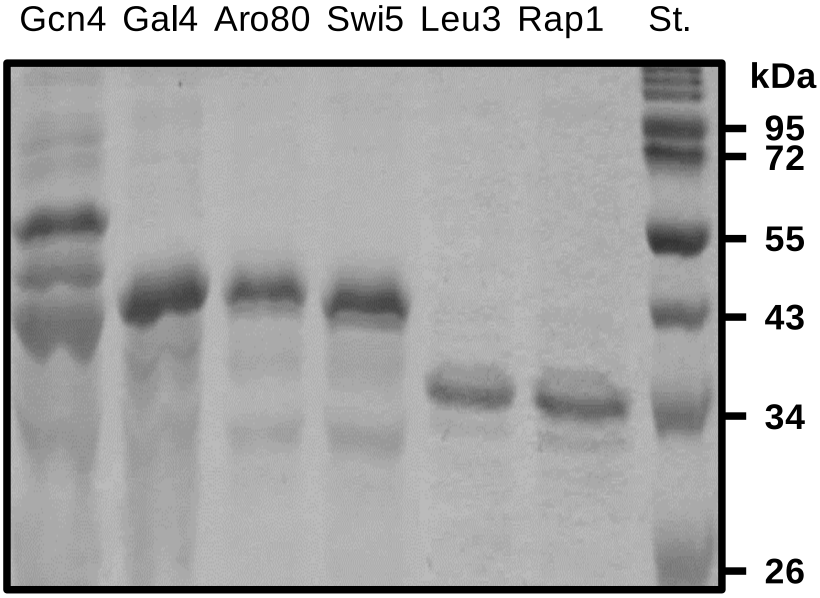

Supplementary Fig. S5: Induction of tac$_{Pr/Op}$-dependent *GST-TAD* fusions by IPTG and affinity purification of GST-TAD fusion proteins using GSH sepharose. **(A)** Bacterial expression plasmids pSH117 (*GST-INO2*$_{1-35}$ TAD1) and pSH118 (*GST-INO2*$_{101-135}$ TAD2) were transformed into *E. coli* strain BL21-CodonPlus(DE3)-RP. Cultures were harvested at the mid-log phase (not induced) or 2 h after addition of

IPTG (induced). Lines 3 show affinity-purified eluates from GSH sepharose. Bacterial protein extracts separated by SDS-PAGE each contained 20 µg of total protein and were stained with Coomassie Brilliant Blue. **(B)** Bacterial expression plasmids pKH60 (*GST-GCN4*$_{9\text{-}172}$), pES20 (*GST-GAL4*$_{768\text{-}881}$), pMG50 (*GST-ARO80*$_{846\text{-}950}$), pDG1 (*GST-SWI5*$_{1\text{-}85}$), pES5 (*GST-LEU3*$_{841\text{-}886}$) and pLJ6 (*GST-RAP1*$_{630\text{-}671}$) were used for affinity purification of GST-TAD fusion proteins. Eluates from GSH sepharose were separated by SDS-PAGE and stained with Coomassie Brilliant Blue.

Supplementary experimental procedure 1 (Enzyme assay of GST):

General information: GST enzymes are able to conjugate glutathione (GSH) with the artificial substrate CDNB (1-chloro-2,4-Dinitrobenzene), leading to a reaction product with high absorbance at 340 nm.

Required solutions:
100 mM CDNB in ethanol;
100 mM reduced GSH in water;
10 x Reaction buffer: 1 M potassium phosphate, pH 6.5

Reaction mixture:

Distilled water:	880 µl
10 x Reaction buffer:	100 µl
CDNB solution:	10 µl
GSH solution:	10 µl
Total:	1000 µl

500 µl of this mixture was transferred to a UV-transparent cuvette and mixed with the GST-containing sample (5-20 µl; sonicated bacterial protein extract from transformants containing GST-TAD fusions). The absorbance at 340 nm was repeatedly recorded for about 5 min. The volume activity of GST was calculated by using the following equation:

$$\Delta A_{340} \,/\, \text{min} \,/\, \text{ml} = \frac{A_{340}\,(t_2) - A_{340}\,(t_1)}{(t_2 - t_1) \times \text{ml sample}}$$
